# Metabolic Redox Coupling Controls Methane Production in Permafrost‐Affected Peatlands Through Organic Matter Quality‐Dependent Energy Allocation

**DOI:** 10.1111/gcb.70390

**Published:** 2025-08-07

**Authors:** John A. Bouranis, Bridget B. McGivern, Ghiwa Makke, Sophie K. Jurgensen, Samantha H. Bosman, Brooke Stemple, Jeffrey P. Chanton, Kelly C. Wrighton, Malak M. Tfaily

**Affiliations:** ^1^ Department of Environmental Science University of Arizona Tucson Arizona USA; ^2^ Department of Soil and Crop Sciences Colorado State University Fort Collins Colorado USA; ^3^ Department of Chemistry and Biochemistry UW Eau Claire Eau Claire Wisconsin USA; ^4^ Department of Earth Ocean and Atmospheric Science Florida State University Tallahassee Florida USA; ^5^ Bio5 Institute, University of Arizona Tucson Arizona USA

**Keywords:** carbon cycling, integrative multi‐omics, metabolomics, metagenomics, metatranscriptomics, methanogenesis, microbial metabolism, peatlands, redox chemistry

## Abstract

Permafrost thaw represents one of Earth's largest climate feedback risks, potentially releasing vast carbon (C) stores as greenhouse gases (GHG). However, our ability to predict emissions remains limited by poor understanding of how changing organic matter (OM) composition affects microbial carbon processing. We test a metabolism‐centered redox framework, which views microbial processes as coupled oxidative‐reductive reactions, to mechanistically explain how organic matter metabolite quality controls greenhouse gas production in permafrost‐affected peatland ecosystems. Rather than relying solely on geochemical redox measurements, our approach examines how microbes balance electron flow through metabolic pathways. Using active layer peat (9–19 cm) from contrasting environments (bog and fen), we employed multi‐omics approaches, including metabolomics, metagenomics, and metatranscriptomics, to link OM chemistry to microbial function. Our results reveal distinct dissolved organic matter metabolite composition, with fen systems enriched in compounds with higher substrate quality (low molecular weight (MW) sugars with high H:C ratios and low aromaticity) and bog systems dominated by compounds with lower substrate quality (high MW phenols with lower H:C ratios and higher aromaticity). In fen samples, these sugar‐like compounds correlated with higher oxidative metabolism and methanogenesis, supported by increased glycolysis gene expression. Initially, electrons from increased oxidative metabolism were balanced through nitrate and sulfate reduction, but as these electron acceptors were depleted, methanogenesis increased to maintain redox balance. Fen samples showed rapid degradation of both high‐ and low‐substrate‐quality compounds, suggesting sufficient energy for efficient C cycling. Conversely, bog samples exhibited more polyphenolic compounds, lower glycolysis activity, and higher stress‐related gene expression, suggesting energy was diverted towards cell maintenance under acidic conditions rather than C processing. This approach suggests that predicting greenhouse gas emissions requires an understanding of how organic matter quality shapes microbial energy allocation strategies, providing a mechanistic framework for improving emission predictions from permafrost‐affected peatlands and similar ecosystems.

## Graphical abstract

Scientists studied how microorganisms in permafrost‐affected peatland ecosystems process organic matter to predict greenhouse gas emissions. Using advanced molecular techniques, they discovered that the chemical quality of organic compounds determines whether microbes produce more or less methane and other greenhouse gases. This research provides a new metabolic framework for predicting carbon emissions from permafrost thaw, one of Earth's largest climate feedback risks, by understanding how microbes balance their energy needs in different environmental conditions.

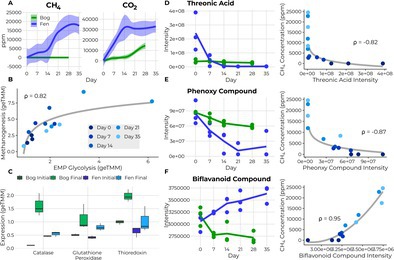

## 1 Introduction

Permafrost‐affected peatlands represent one of Earth's largest vulnerable carbon sinks, storing approximately twice as much carbon as the atmosphere. As climate change accelerates permafrost thaw, these ecosystems are predicted to shift from carbon sinks to sources, amplifying a potentially devastating climate feedback loop (Schuur et al. 2015, 2022). Thawing fundamentally alters organic matter composition through two mechanisms: (1) liberation of ancient, selectively decomposed carbon, and (2) input of new, labile carbon from enhanced plant productivity (Hodgkins et al. 2014; Holmes et al. 2022; Wilson et al. 2022). These shifts reshape microbial communities and their metabolism (Freire‐Zapata et al. 2024; Woodcroft et al. 2018), yet the mechanistic links between organic matter quality and microbial carbon processing remain poorly understood.

In previous work (Bouranis and Tfaily 2024), we developed a microbial metabolism‐centered redox framework that views microbial activity in anaerobic environments, such as water‐saturated peatlands, as a balance between interconnected oxidative and reductive processes. The predominantly anaerobic conditions in peatlands make this redox‐coupling framework particularly relevant, as microbial communities must maintain electron balance without oxygen as the terminal electron acceptor. Unlike traditional geochemical redox studies that focus on redox potential (Eh) or speciation, our approach reframes redox as a dynamic microbial process involving coupled oxidative and reductive reactions. During oxidative metabolism, electrons are removed from substrates and transferred to electron carriers, while in reductive metabolism, these electrons are transferred to organic or inorganic compounds, coupled with adenosine triphosphate (ATP) production through fermentation or respiration. These two processes are interdependent—oxidation must be balanced by reduction to maintain electron flow.

Across anaerobic systems, increased terminal electron acceptor (TEA) availability can stimulate oxidative metabolism, though responses vary by environment. For example, sulfate addition enhanced carbon oxidation in marine sediments (Canfield et al. 1993), while TEA amendments in peatlands showed variable outcomes for carbon mineralization (Keller and Bridgham 2007). Where preferred TEAs are limited, alternative redox pathways emerge, such as fermentation, organic electron acceptor use, or syntrophic partnerships involving hydrogen/formate exchange (Klüpfel et al. 2014). This has been observed in ruminants, where methanogenesis inhibition leads to upstream accumulation of metabolic products and reduced sugar degradation (Ungerfeld 2015). In peatlands, humic substances can serve dual roles as electron donors and acceptors, further complicating redox dynamics and carbon mineralization (Lipson et al. 2010; Keller and Bridgham 2007). Thus, the relationship between TEAs and carbon oxidation is context‐dependent rather than universal.

Our framework could advance the understanding of climate‐carbon feedbacks by linking substrate composition directly to microbial function and greenhouse gas production, something thermodynamic models alone cannot do (Schmidt et al. 2011; Sinsabaugh et al. 2013). Traditional thermodynamic models identify reaction favorability but not actual metabolic activity (Jin and Bethke 2007; LaRowe and Van Cappellen 2011). We hypothesize that our framework can connect organic matter quality to metabolic electron transfers, potentially offering mechanistic insights when paired with multi‐omics data. Higher substrate quality—defined by low molecular weight, high H:C ratios, and low aromaticity—tends to support increased microbial activity, as degradation of labile compounds requires less energy and yields greater net return (LaRowe and Van Cappellen 2011; Kleber et al. 2011). In redox terms, high‐quality substrates enhance oxidative metabolism and generate more electrons that must be balanced via reduction. Microorganisms use different glycolytic pathways depending on substrate availability: the EMP pathway yields more ATP and NADH per glucose molecule, favoring energetically rich conditions, while the ED pathway produces NADPH and is advantageous under nutrient stress or oxidative pressure (Conway 1992; Flamholz et al. 2013). Thus, glycolytic preferences reflect metabolic adaptations to environmental conditions. However, substrate quality alone does not determine microbial strategy. In complex environments like permafrost‐affected peatlands, other factors influence energy allocation: pH stress, temperature limitations, nutrient availability, and the presence of inhibitory compounds like phenolics. These co‐occurring constraints may suppress methanogenesis despite the thermodynamic favorability of the process.

To test this framework, we focus on Stordalen Mire in northern Sweden, a globally significant site for studying permafrost thaw. Warming has created contrasting habitats within the mire—acidic, *Sphagnum*‐dominated bogs and neutral, sedge‐rich fens—with stark differences in greenhouse gas emissions (Hodgkins et al. 2014; Holmes et al. 2022). Bogs primarily emit CO_2_, while fens emit both CO_2_ and CH_4_. While these patterns are well documented (Cory et al. 2022; Holmes et al. 2022), their underlying metabolic causes remain unclear.

Multiple hypotheses have been proposed to explain these bog‐fen GHG differences, including TEA recycling (Lipson et al. 2010), methane oxidation (Singleton et al. 2018), methanogen community structure (Woodcroft et al. 2018), pH‐driven suppression of methanogenesis (Ye et al. 2012), substrate competition (Pester et al. 2012), and plant‐mediated differences in organic matter (Hodgkins et al. 2014). Phenolic compound accumulation in bogs may also inhibit microbial activity (Wang et al. 2015). While informative, these studies often examine single variables in isolation, limiting mechanistic insight into how multiple interacting factors shape metabolism.

We propose that our redox framework could address this gap by integrating organic matter chemistry, environmental stressors, and microbial energetics. By linking organic matter quality to microbial energetics, our framework could explain why thermodynamically favorable processes like methanogenesis may not occur under certain environmental pressures. We test three key hypotheses: (1) Bog and fen systems will exhibit distinct organic matter composition, with bogs dominated by recalcitrant compounds and fens enriched in more bioavailable substrates; (2) Energy allocation strategies differ between systems—fen microbes will prioritize methane production for redox balance, while bog microbes will divert energy toward stress responses due to acidic conditions (pH 3.5–4.5) and low substrate quality; (3) These compositional and energetic differences will create distinct redox pressures, with readily available substrates in fen peat driving higher oxidative metabolism that requires methanogenesis for redox balance, while bog conditions suppress methanogenesis despite thermodynamic favorability.

In acidic conditions (pH 3.5–4.5), microorganisms may invest energy in stress responses such as pH homeostasis, membrane stabilization, and protein repair (Patzner et al. 2020; Fernandez‐Calvino and Bååth 2010), leaving less available energy for methanogenesis. Stress‐induced energy tradeoffs are known to affect carbon flow (Dijkstra et al. 2011; Schimel et al. 2007), but their impact on redox balance in thawing peatlands remains poorly understood.

To test this hypothesis, we conducted nutrient‐amended anoxic microcosm incubations using the active layer peat (9–19 cm depth) from bog and fen habitats at Stordalen Mire. Nutrient amendments eliminated nutrient limitation as a confounding factor, allowing us to isolate the role of organic matter quality and redox pressure in shaping microbial metabolism and greenhouse gas production. We employed a multi‐omics approach that provides complementary insights into the relationships between organic matter quality and microbial metabolism: (1) Metabolomics (LC–MS/MS) characterizes organic matter/metabolite composition, allowing us to identify differences in substrate availability and transformation between bog and fen peat. (2) Metagenomics reveals the metabolic potential of microbial communities, identifying key pathways for carbon processing and electron transfer. (3) Metatranscriptomics shows which pathways are actively expressed, providing real‐time insights into microbial responses to substrate availability and redox conditions. This multi‐omics approach, combining genome‐resolved metatranscriptomics and metabolomics, allows us to directly link organic matter quality to microbial metabolism and GHG production.

If our metabolism‐centered redox framework hypothesis is correct, we expect to observe (i) higher expression of oxidative pathways in fen peat corresponding with higher substrate quality; (ii) balanced expression of reductive pathways (including methanogenesis) in fen peat to maintain redox equilibrium; and (iii) energy allocation toward stress responses in bog peat at the expense of methanogenesis. The integration of these three omics approaches provides unprecedented resolution into the mechanisms linking organic matter quality to greenhouse gas emissions in these contrasting peatland habitats.

This study aims to provide critical insights into how organic matter composition controls greenhouse gas emissions through microbial metabolic regulation. Understanding these relationships is essential as Arctic ecosystems undergo unprecedented transitions due to climate change. Our findings may have implications beyond permafrost regions, offering new approaches for predicting and potentially mitigating methane emissions/production in other systems, from wetlands to agricultural settings. The metabolism‐centered redox framework, by mechanistically linking substrate availability to metabolic outcomes, addresses key uncertainties in current climate projections. This work bridges molecular microbial ecology with ecosystem‐scale biogeochemistry, advancing our ability to predict and manage carbon‐climate feedbacks in a warming world.

## 2 Materials and Methods

### 2.1 Peat Sampling

Peat cores were collected from a fen and a bog in Stordalen Mire (Abisko, Sweden; 68° 22′ N, 19° 03′ E) in July 2016 using an 11‐cm‐diameter push corer. The cores were sectioned in the field, and the 9–19 cm depth section from within the active layer overlying permafrost was selected for this study. Each sectioned sample was immediately sealed in a plastic bag. Cores were transported on ice from the field to the research station and stored at −20°C until shipment on dry ice, then returned to −20°C storage until microcosm construction.

### 2.2 Incubation Setup

Incubations were set up as previously described (McGivern, Ellenbogen, et al. 2024). To construct the incubation microcosms, the frozen active layer peat was thawed at room temperature for 1 h. Roughly 6 g of thawed fen and bog peat were added to sterilized glass Balch tubes in triplicate. The tubes were then sealed with a sterile butyl stopper and aluminum crimp. Tubes were flushed with N_2_ gas for 5 min, then 11 mL of sterile anoxic media was added. The media consisted of (per liter) 0.25 g ammonium chloride, 0.60 g sodium phosphate, and 0.10 g potassium chloride in sterile water with N_2_ headspace. These concentrations (approximately 4670 μM NH_4_^+^, 3660 μM PO_4_^3−^, and 1340 μM K^+^) are substantially higher than typical porewater concentrations found in natural peatlands to ensure non‐limiting nutrient conditions for our experimental objectives. For context, measurements from the same Stordalen Mire field site reported by Emerson et al. (2018) showed porewater ammonium concentrations ranging from 0.19 to 21.14 μM in bog environments (with most values between 0.5 and 5 μM) and from 0 to 23.65 μM in fen environments, with greater variability at depth. Phosphate concentrations in bog environments ranged from 5.00 to 283.8 μM, with most values between 50 and 120 Μm (unpublished data). These experimental nutrient concentrations are intentionally elevated compared to field conditions, as is common practice in laboratory incubation studies designed to eliminate nutrient limitation as a confounding factor.

Tubes were then vortexed to create a slurry and flushed with N_2_ gas for 10 more minutes. The day 0 microcosms were immediately harvested, and the remaining tubes were placed in a dark incubator at 19°C, representing the field temperature of the active layer during summer months in this permafrost‐affected ecosystem (Fofana et al. 2022). Microcosms were then sacrificially sampled at various timepoints. Both bog and fen peat microcosms were harvested in triplicate at days 0, 7, and 14. We selected the final sampling time point based on carbon dioxide/methane production: day 28 for bog samples and day 35 for fen samples, respectively. To destructively sample the microcosms, headspace gas was first taken from the tubes (described below, Gas Measurements). Then, tubes were uncapped, and the slurry was decanted into a 15 mL Falcon tube and centrifuged for 10 min at 16,000× *g*. From the clarified supernatant, 1 mL aliquots were collected from each of the triplicate samples at each time point (3 replicates × 4 time points for each habitat type) and stored at −80°C in 1.7 mL microcentrifuge tubes for LC–MS/MS metabolome analysis. The remaining liquid was discarded, and the pellet was immediately stored at −80°C for nucleic acid extraction for DNA and RNA.

Our nutrient‐amended incubation methodology differs from previous approaches focused primarily on dissolved organic matter (DOM) characterization. Unlike previous studies that extracted porewater directly from the field (AminiTabrizi et al. 2020) or used incubations with milli‐Q water (Hodgkins et al. 2014), we utilized a nutrient‐rich media to promote microbial activity and metabolic processes. This approach allowed us to observe significant microbial dynamics, including substantial methane emissions, extensive microbial decomposition, and rapid consumption of organic matter, providing insights into the metabolic potential of these communities under non‐limiting nutrient conditions.

### 2.3 Gas Measurements

To measure headspace CO_2_ and CH_4_ concentrations, 10 mL of headspace was removed from the microcosms being harvested using a gas‐tight Hamilton syringe and stored in glass 6.9 mL Exetainer vials. Vials were stored at room temperature until the end of the experiment and shipped at ambient temperature to Florida State University for GC‐FID analysis. CO_2_ and CH_4_ were measured on a Shimadzu 8A gas chromatograph with a carbosphere packed column operated at 140°C. CO_2_ was converted to CH_4_ for flame ionization analysis (FID) by running it across a methanizer. Samples were quantified relative to calibrated air gas standards. Total CO_2_ and CH_4_ concentrations were calculated by accounting for both gaseous and dissolved phases, following the approach described in Hodgkins et al. (2014). Dissolved CO_2_ (including carbonic acid, bicarbonate, and carbonate species) and dissolved CH_4_ were calculated using Henry's Law constants adjusted for temperature and pH, with the measured headspace concentrations used to determine the corresponding dissolved concentrations. The sum of gaseous and dissolved phases represents the total production of each gas. See Table S1 for CO_2_ and CH_4_ data.

### 2.4 Metabolomics Data Generation and Cleaning

A 1 mL aliquot of active layer peat microcosm supernatant was thawed at 4°C. Following thawing, the sample was centrifuged to remove any particles that may have formed during the freeze–thaw process. Next, each sample was split into two vials (0.5 mL each), one for hydrophilic interaction liquid chromatography (HILIC) and the other for reverse‐phase (RP) liquid chromatography as described previously (Portman et al. 2024; Wyatt et al. 2024). Samples in both vials were dried down using a Vacufuge plus (Eppendorf, USA). Following this, the samples were resuspended in 350 μL of either a 50% acetonitrile/50% water solution for HILIC or an 80% water/20% HPLC‐grade methanol solution for RP.

Metabolomics data was generated at the University of Arizona Analytical & Biological Mass Spectrometry Facility. A Thermo Scientific Vanquish Duo ultra‐high performance liquid chromatography system (UHPLC) was used for liquid chromatography. Extracts were separated using a Waters ACQUITY HSS T3 C18 column for RP separation and a Waters ACQUITY BEH amide column for HILIC separation. 1 μL volume of each extract was eluted from the column as follows: for RP, the gradient went from 99% mobile phase A (0.1% formic acid in H_2_O) to 95% mobile phase B (0.1% formic acid in methanol) over 16 min. For HILIC the gradient went from 99% mobile phase A (0.1% formic acid, 10 mM ammonium acetate, 90% acetonitrile, 10% H_2_O) to 95% mobile phase B (0.1% formic acid, 10 mM ammonium acetate, 50% acetonitrile, 50% H_2_O). Both columns were run at 45°C with a flow rate of 300 μL/min.

Spectral data was collected using Thermo Scientific Orbitrap Exploris 480. The instrument operated with a spray voltage of 3500 V for positive mode (for RP) and 2500 V for negative mode (for HILIC) using the H‐ESI source. Both the ion transfer tube and vaporizer temperature were 350°C. Compounds were fragmented using data‐dependent MS/MS with HCD collision energies of 20, 40, and 80. Compound Discoverer 3.3.2.31 software (Thermo Fisher Scientific) was used to analyze the data using the untargeted metabolomics workflow. Briefly, the spectra were first aligned, followed by a peak‐picking step. Metabolite annotation was performed using an in‐house mzVault database built using 1200 reference standards, spectral libraries, and compound databases. First, fragmentation scans, retention time, and ion mass of unknown compounds were compared with those in the in‐house database. For searches against the in‐house mzVault library, a spectral similarity score threshold of 50% was applied. Second, fragmentation scans (MS2) searches in mzCloud were performed, which is a curated database of MSn spectra containing more than 9 million spectra and 20,000 compounds. For mzCloud searches, a spectral similarity score threshold of 60% was applied. The structure‐matching module of SIRIUS software was used to complement MS2 annotations determined by mzCloud (J. Wang, n.d.). Third, predicted compositions were obtained based on mass error, matched isotopes, missing number of matched fragments, spectral similarity score (calculated by matching theoretical and measured isotope patterns), matched intensity percentage of the theoretical pattern, the relevant portion of MS, and the MS/MS scan. The spectral similarity score was calculated based on the match between the theoretical and measured isotope patterns, with an intensity tolerance of 30%. Predicted compositions were assigned based on a mass tolerance of 5 ppm, a signal‐to‐noise threshold of 3, and a minimum spectral fit of 30% with fragment matching mass tolerance of 5 ppm. Retention time was used for compound identification in mzVault but was not directly used for elemental composition assignments.

To increase annotation coverage, the Canopus module of SIRIUS software was used to predict compound class based on MS2 spectra (Böcker et al. 2009; Dührkop et al. 2019). The compound level of annotation was assigned according to the Metabolomics Standards Initiative (Sumner et al. 2007) as follows: Level 1: compounds with an exact match to a standard reference compound in our in‐house mzVault library; Level 2: compounds with a full match to online spectral databases using mzCloud or other MS2 databases (based on MS2 spectra matching); and Level 3: compounds with only a molecular formula (generated through the Predicted Compositions node function available in Compound Discoverer).

To reduce noise and collinearity and remove artifactual peaks from the dataset, a custom in‐house script was used to identify source fragments and ions resulting from neutral mass loss. Briefly, ions were first binned based on their retention time using a retention time tolerance window of 0.005 s. Next, compounds that co‐eluted were examined for correlations in their raw intensity, and only ions that had a linear correlation across all samples greater than 0.98 were retained. The MS2 spectra of the heaviest ion (the parent ion) were searched for each candidate fragment ion with mass similarity of less than 5 ppm. Only candidate fragment ions present in the MS2 of the parent ion were retained. Lastly, the resulting candidate list was manually inspected through MS2 matching, MS2 annotation (based on neutral mass losses), and by examining the correlation patterns to determine the validity of the candidate fragment ions. Validated fragment ions were removed from the dataset. Additionally, compounds with predicted compositions containing halogen ions were removed from the dataset, as it is unlikely these compounds would exist naturally in active layer peat soils and are thus likely artifacts of analysis. Metabolomics data is available in Table S2.

### 2.5 Metabolomics Statistical Analysis

To handle the large range of values (peak intensities) in our LC‐MS/MS metabolomics dataset, log transformation and Pareto scaling were used to transform the data and make it suitable for downstream statistical analysis. To handle 0 s in the data, a generalized log transform was applied using the formula *x* = (√(*x* ^2^ + a^2^))/2 where *x* represents each individual metabolite intensity value in our sample matrix, and a is the minimum non‐zero value identified across all spectra collected in the project. After log transformation, Pareto scaling was applied across the entire dataset by dividing each metabolite's values by the square root of the standard deviation of that metabolite across all samples. This approach reduces the relative importance of large values while keeping the data structure partially intact (Van den Berg et al. 2006). All three biological replicates for each condition (bog and fen) and time point were included in this transformation and scaling process, preserving the biological variability between replicates. This approach ensures that values remain comparable across multiple metabolites and samples by using a single value (also called lambda) that determines how much the data is compressed or stretched during transformation. The result is a normalized intensity matrix with the same dimensions as the original intensity matrix.

To examine drivers of variation and large‐scale patterns in the metabolomics data, a principal component analysis (PCA), as implemented in the R package mixOmics (v6.24.0) (Rohart et al. 2017), was used. To identify metabolomic features significantly different between the bog and the fen, a linear model was fit for each metabolomic feature. The peak intensity was used as the dependent variable, with the day of incubation, sample type (bog or fen), and their interaction as the independent variables. The interaction term allows us to assess how the effect of sample type on metabolomic features varies across different days of incubation, thereby providing insight into whether the relationship between sample type and metabolite levels changes over time. Contrasts, as implemented by the R package multcomp (v1.4‐25) (Hothorn et al. 2002), were used to find features significantly different between the bog and the fen at each timepoint. Multiple hypothesis testing was corrected using the Benjamini–Hochberg method (Benjamini and Hochberg 1995). To examine class‐wise differences between bog and fen, chemRICH analysis (Barupal and Fiehn 2017) were used. Metabolites associated with methane production was determined using the WGNCA package in R (v1.72‐1) (Langfelder and Horvath 2008). WGCNA analysis was conducted using log‐transformed and pareto‐scaled metabolomics data, a soft‐thresholding power of 14, and a signed network. Pearson's correlation was used to determine relationships between eigengene values and methane emissions. To aid in interpretability, metabolomics features without MS2 annotations were removed, and only the top 100 most strongly correlated features for both the positive and negative modules were retained. To further validate relationships, Spearman's correlations between raw metabolomic feature intensity and methane emissions were compared. The nominal oxidation state of carbon (NOSC) for selected compounds was calculated using the formula: $\text{NOSC}=-\left(\frac{-Z+4C+H-3N-2O+5P-2S}{C}\right)$ (LaRowe and Van Cappellen 2011). All data visualization was created using R packages ggplot2 (v3.5.1) (Wickham 2011) and cowplot (v1.1.1) (Wilke 2015).

### 2.6 RNA and DNA Extraction

Active layer fen and bog DNA were extracted from the frozen pellets using the ZymoBIOMICS DNA/RNA Miniprep Kit. For the lysis step, the pellet was resuspended in 750 μL of the kit lysis solution, transferred to the provided lysis bead tubes, and lysed with a FastPrep‐24 at 5 m/s for 20 s. We continued with the combined RNA extraction for the fen samples. Bog RNA was extracted from the frozen pellets using the Zymo Quick‐RNA Microbe Microprep kit according to the manufacturer's instructions. Extracted DNA and RNA were quantified using the Qubit HS dsDNA and HS RNA kits, respectively. Extracted DNA was stored at −20°C until sequencing, and extracted RNA was stored at −80°C until sequencing.

### 2.7 Metagenome Sequencing, Assembly, and Binning

We constructed a metagenome‐assembled genome (MAG) database as described in McGivern, Ellenbogen, et al. (2024). Briefly, metagenomes were obtained from 2 active‐layer peat bog microcosms (day 0 and day 28) and 4 active‐layer fen microcosms (day 0, day 7, day 14, and day 35), in addition to six other microcosms using Stordalen Mire fen and bog peat not described here. These additional six samples were used purely for metagenome sequencing and genome recovery. Metagenome libraries were prepared using the Tecan Ovation Ultralow V2 DNA‐Seq kit and sequenced on a NovaSeq6000 system (Illumina, v.1.5 chemistry, S4 flow cell, 2 × 150 bp) at the Genomics Shared Resource Facility at the University of Colorado Anschutz Medical Campus.

Fastq files were trimmed with sickle (v1.33) (Joshi and Fass 2011). Individual assemblies, co‐assemblies, and iterative assemblies were conducted (1) MEGAHIT (v1.2.9) (Li et al. 2015) using the following flags: ‐‐k‐min 31 ‐‐k‐max 121 ‐‐k‐step 10. Coverage information was determined for contigs > 2500 base pairs using bbmap (Bushnell 2014) and a BAM file was generated using samtools (v1.9) (Li et al. 2009). These contigs were binned using metaBAT2 (v1.2.9) (Kang et al. 2019). MAG quality was assessed using checkM2 (v0.1.3) (Chklovski et al. 2023). MAGs with completion > 50% and contamination < 10% were retained as medium and high‐quality (MQ/HQ) MAGs (Bowers et al. 2017).

The MQ/HQ MAGs recovered from these efforts were dereplicated at 99% nucleotide identity with 13,290 MAGs from Stordalen Mire (McGivern, Cronin, et al. 2024). MAG taxonomy was inferred using the Genome Taxonomy Database Toolkit (GTDB‐tk v2.3.0 r214) (Chaumeil et al. 2022). MAGs were annotated using DRAM (v1.4.4) (Shaffer et al. 2020) and CAMPER (v1) (McGivern, Cronin, et al. 2024). The MAGs that recruited reads in the active layer peat bog and fen microcosms and their annotations are provided in Tables S3 and S4.

### 2.8 Metatranscriptome Sequencing and Analysis

Fen metatranscriptome libraries were prepared at the Joint Genome Institute as described in McGivern, Ellenbogen, et al. (2024). Briefly, plate‐based RNA sample prep was performed on the PerkinElmer Sciclone NGS robotic liquid handling system using the FastSelect 5S/16S/23S for bacterial rRNA depletion kit (Qiagen) with RNA blocking oligo technology to block and remove rRNA from 100 ng of total RNA input. An Illumina sequencing library was then created from the fragmented and rRNA‐depleted RNA using the TruSeq Stranded Total RNA HT sample prep kit (Illumina) following the protocol and with 10 cycles of PCR for library amplification. The prepared libraries were quantified using KAPA Biosystems' next‐generation sequencing library qPCR kit and run on a Roche LightCycler 480 real‐time PCR instrument. Sequencing of the flow cell was performed on the NovaSeq sequencer (Illumina) using NovaSeq XP V1.5 reagent kits, S4 flow cell, following a 2 × 151 indexed run recipe.

Bog metatranscriptome libraries were prepared at the Genomics Shared Resource Facility at the University of Colorado Anschutz Medical Campus. The Zymo‐Seq RiboFree Total RNA Library Kit was used to prepare the sequencing library. Libraries were sequenced on an Illumina NovaSeq 6000 system (v.1.5 chemistry, S4 flow cell, 2 × 150 bp).

Raw bog and fen metatranscriptome reads were quality trimmed and adapters removed using bbduk (Bushnell 2014) with the following flags: *k* = 23, mink = 11, hdist = 1, qtrim = rl, trimq = 20, minlength = 75 (Table S3). Each sample was randomly subsampled to 50,000,000 pairs of trimmed reads, and the reads were mapped against the database of 99% dereplicated MAGs using Bowtie2 (v2.4.5) (Langmead and Salzberg 2012) with the following flags: ‐D 10 ‐R 2 ‐N 1 ‐L 22 ‐i S,0,2.50. The output SAM file was converted to BAM using samtools and filtered using the reformat.sh script in the bbtools package using: idfilter = 0.97, pairedonly = t, and primaryonly = t.

Processed metatranscriptomics data was normalized using geTMM for comparison across samples (Smid et al. 2018). The metatranscriptome mapping is given in Table S4. Pathways were mapped using the logical expression as defined by KEGG. Briefly, enzymes that exist in a complex, as denoted by a plus sign in the logical expression, were averaged, while those that are alternatives, as denoted by a comma, were summed. All steps were averaged across a pathway. Pathway definitions were adapted from KEGG to minimize overlap between pathways. Pathways are presented in Table S5.

### 2.9 Metatranscriptome Curation

The proportion of MAGs encoding for a metabolic pathway was determined using the annotation from DRAM. A MAG was considered active if the total transcript count across all genes in a sample was greater than 15. For MAGs that were actively transcribing, they were considered to have a pathway encoded if they contained gene copies for at least 60% of the steps in a metabolic pathway. Differences in genetic encoding between the active layer peat bog and fen were determined using the Wilcoxon test.

## 3 Results

### 3.1 Fen and Bog Active Layer Peat Have Distinct Organic Matter Metabolite Composition

**FIGURE 1:**
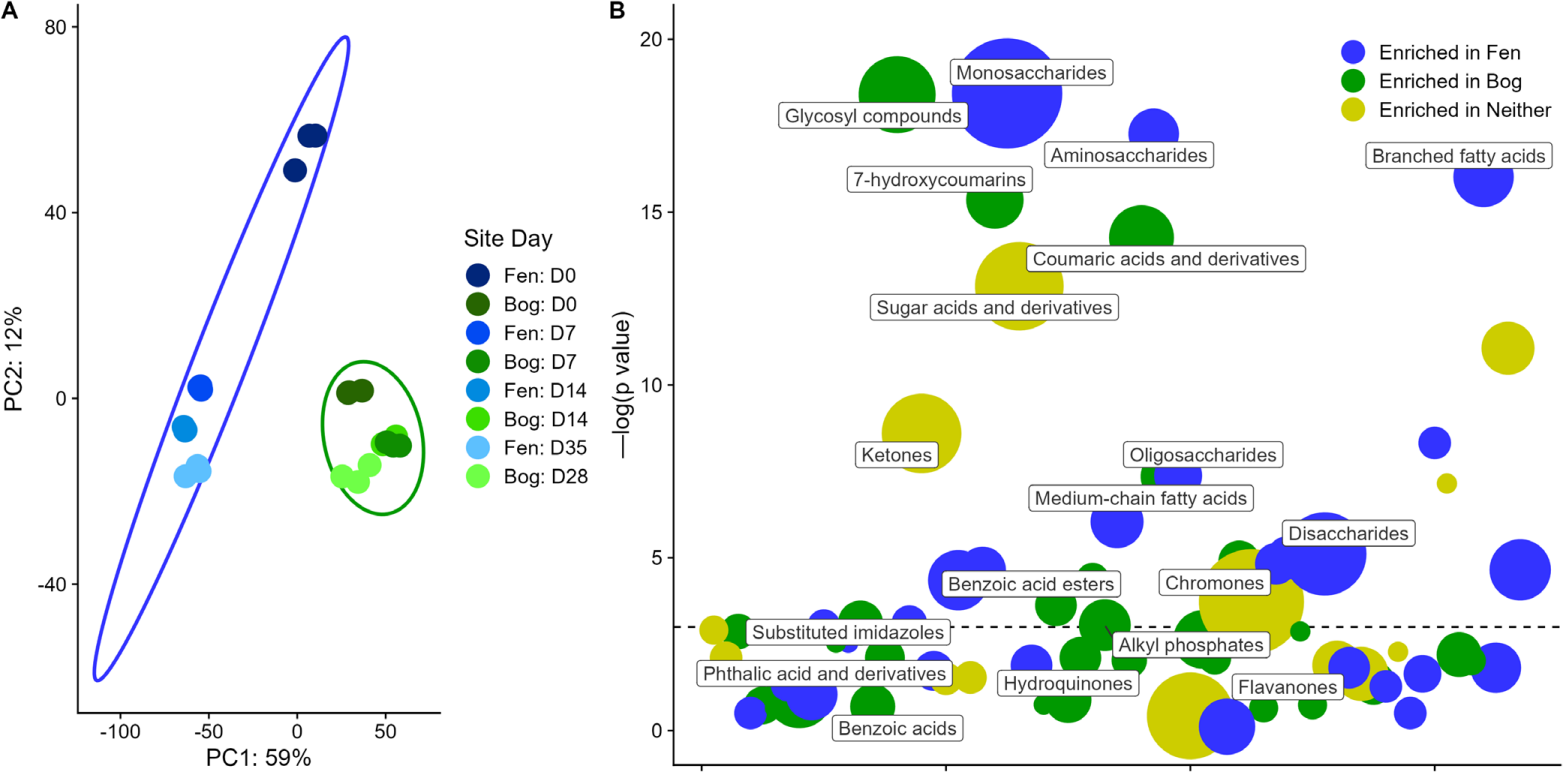
Metabolite composition differs between bog and fen microcosms as revealed by LC–MS/MS. (A) PCA plot of metabolites as determined by LC–MS/MS. Ellipses represent bog and fen samples. D0, 7, 14, 28, and 35 represent days of nutrient‐amended incubation. Separation between bog and fen demonstrates that the two have distinct metabolomic profiles. (B) Chemical classes enriched in bog and fen as determined by LC–MS/MS. The *y*‐axis indicates how significantly enriched a class of compounds is and the bubble size indicates the number of compounds in that class. Bubble color represents which microcosm that class of compounds is enriched in green for bog or blue for fen. The *x*‐axis is arbitrary. Bubbles above the dashed line have a *p*‐value less than 0.05. Blue represents fen samples and green represents bog samples.

Our metabolism‐centered redox framework (Bouranis and Tfaily 2024) proposes that organic matter composition drives ecosystem‐level carbon cycling by controlling substrate availability and oxidative pressure. We characterized DOM metabolite composition in the supernatants of the active layer peat bog and fen nutrient‐amended microcosms from Stordalen Mire, a permafrost‐affected ecosystem, where previous studies have documented contrasting vegetation, hydrology, and pH conditions between fen and bog habitats (Hodgkins et al. 2014; Hough et al. 2022; Wilson et al. 2022). Principal component analysis of metabolomics data generated via LC–MS/MS showed distinct metabolomes for active layer peat bog and fen microcosms (Figure 1A). Importantly, the fen metabolome displayed greater temporal variation across the incubation period, indicating more dynamic carbon processing compared to the relatively static bog metabolome. Chemical class enrichment analysis (Barupal and Fiehn 2017) (Figure 1B and Table S6) revealed contrasting metabolic environments (Fudyma et al. 2019; Holmes et al. 2022). Fen microcosms were enriched in readily oxidizable sugar‐like substrates (amino saccharides, mono‐, di‐, and oligosaccharides) that can drive high oxidative pressure (e.g., Lin et al. 2019). These readily oxidizable substrates provide electrons through oxidation reactions that must be balanced through reductive pathways, consistent with observations that these compounds often support fermentative metabolism when oxygen is limited (Vasco‐Correa et al. 2018; Smith et al. 2019). During fermentation of these carbohydrates, NAD+ is regenerated through the reduction of organic compounds, producing alcohols and organic acids as terminal electron acceptors without the involvement of an electron transport chain (Hoelzle et al. 2014).

In contrast, bog microcosms contained compounds that typically constrain oxidative metabolism: medium‐chain fatty acids, chromones, coumaric acids, 7‐hydroxycoumarins, and glycosyl compounds. Previous studies have demonstrated that medium‐chain fatty acids can inhibit respiratory metabolism by disrupting membrane integrity (Wilson et al. 2010), while phenolic compounds such as coumaric acids and chromones have been shown to act as antioxidants by scavenging reactive oxygen species (ROS) and inhibiting oxidative enzymes (Pękal and Pyrzynska 2014; Rice‐Evans et al. 1996). These compounds may create conditions that favor alternative redox balancing strategies, such as the Entner–Doudoroff pathway, which generates NADPH directly from glucose oxidation and offers advantages in managing oxidative stress (Klingner et al. 2015).

These distinct chemical compositions suggest fundamentally different metabolic landscapes, with fen environments creating higher oxidative pressure that requires balanced reductive metabolism. The subsequent sections explore how these differences in substrate availability influence microbial metabolism and greenhouse gas production (Fudyma et al. 2019; Holmes et al. 2022).

### 3.2 Substrate Quality Drives Contrasting Energy Allocation Strategies Between Active Layer Peat From Bog and Fen Ecosystems

Next, we examined how the distinct DOM metabolite compositions influence microbial community composition and metabolic strategies in active layer peat bog and fen microcosms. Genome‐resolved metatranscriptomics revealed that 998 MAGs recruited transcripts in bog microcosms versus 1899 in fen microcosms, with 877 MAGs shared between ecosystems. *Acidobacteriota* dominated both habitats, representing 32% of active MAGs in bog and 23% in fen, followed by *Pseudomonadota* (22% in bog, 16% in fen) (Figure S1A).

Next, we examined the proportion of transcripts detected belonging to each phylum to gain insight into the functional differences between the two communities (Figure S1B). Transcript allocation revealed significant functional differences between communities. In bog microcosms, *Acidobacteriota* and *Pseudomonadota* dominated expression (64% and 29% of transcripts, respectively). The fen showed a more balanced expression across phyla: *Pseudomonadota* (26%), *Bacteroidota* (22%), *Bacillota_A* (16%), *Acidobacteriota* (7%), *Chloroflexota* (6%), and *Actinomycetota* (6%) (Figure S1B). Notably, methanogens represented 2% of the fen metatranscriptome compared to only 0.3% in bog, consistent with expected differences in methane production.

**FIGURE 2:**
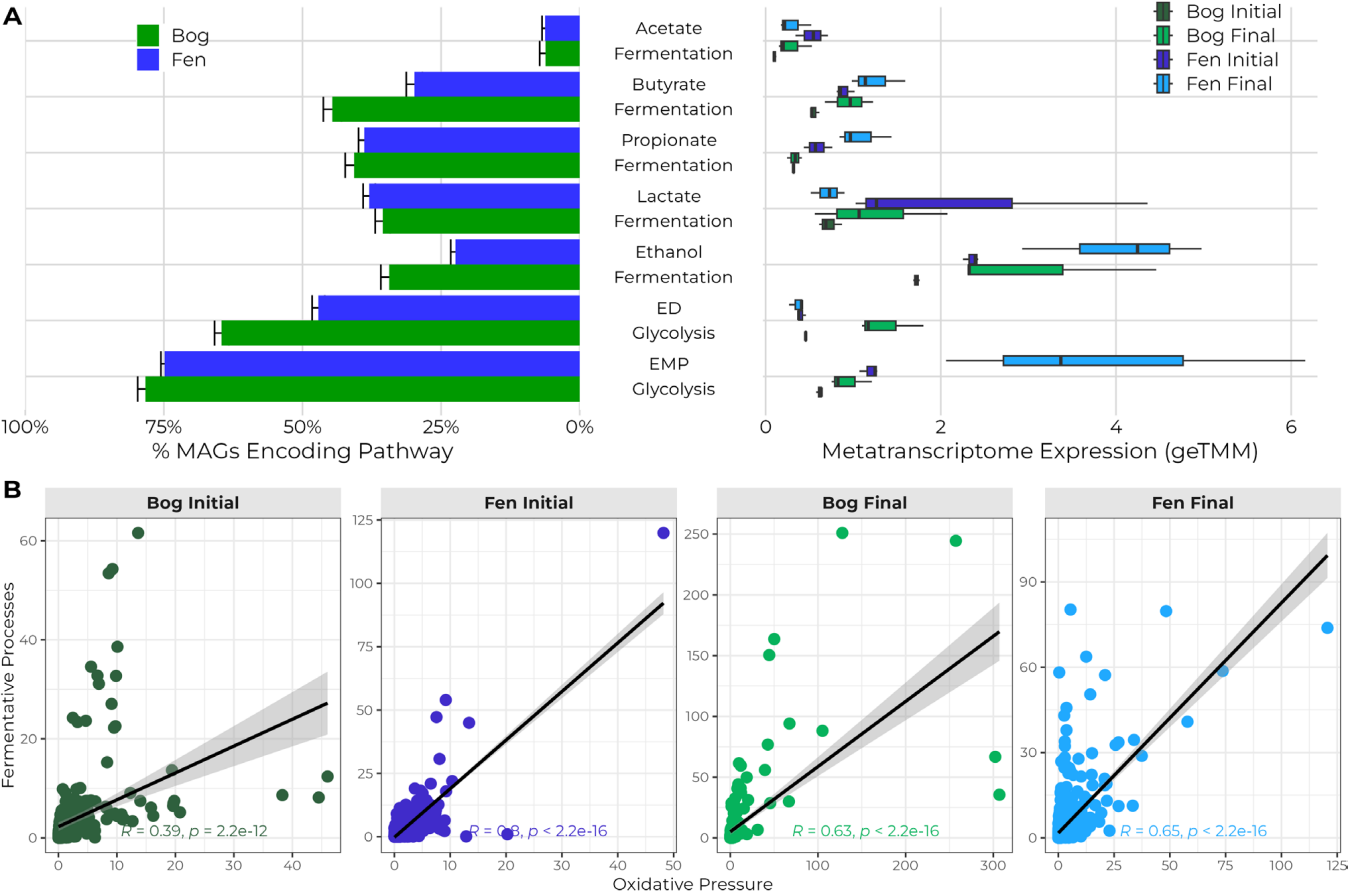
(A) Metabolic pathway encoding and expression differences between bog and fen microcosms. The proportion of metagenome‐assembled genomes (MAGs) that encode for a given metabolic pathway out of all MAGs which recruited transcripts for each microcosm habitat is shown on the left‐hand side of the plot. This indicates the genetic potential for each pathway within the active microbial community. The right‐hand side of the plot shows the mapped transcription of that pathway in each sample and timepoint, representing actual metabolic activity. Pathways are organized into oxidative metabolism (EMP‐glycolysis, ED‐glycolysis) and reductive metabolism (various fermentation pathways), illustrating the balance between electron‐generating and electron‐consuming processes in each environment. Bog and fen initial refer to day 0 while bog final refers to day 28 and fen final refers to day 35. Note the contrasting patterns between genetic potential (MAG proportion) and actual expression, particularly for the bog samples, supporting our hypothesis that substrate availability rather than genetic capacity drives metabolic activity differences between these environments. (B) Correlation between oxidative metabolism (ED + EMP‐glycolysis) and fermentation processes at the MAG level for each timepoint. Each point represents an individual MAG, with oxidative pathway expression on the *x*‐axis and fermentation pathway expression on the *y*‐axis. Pearson correlation coefficients (*r*) show distinct temporal patterns: bog exhibits a strengthening positive correlation over time (*r* = 0.39 to *r* = 0.63), while fen displays a weakening correlation (*r* = 0.8 to *r* = 0.65). These differing correlation patterns reveal contrasting metabolic strategies, with bog MAGs developing tighter coupling between oxidative and reductive processes under substrate limitation, while fen MAGs show decreasing coordination as alternative electron‐accepting processes become more prominent.

To understand how substrate availability affects metabolism, we focused on carbohydrate degradation pathways, given their importance in peatlands and the increased availability of sugars in the fen (Woodcroft et al. 2018) (Figure 2). While the fen showed consistently higher expression of EMP‐glycolysis throughout the incubation compared to the bog (Figure 2A, right panel), both habitats exhibited an increased pathway expression at their respective final sampling points relative to earlier time points, though with different magnitudes of change. This temporal shift across the incubation period suggests a dynamic response to changing conditions during incubation—possibly due to the breakdown of complex compounds into more bioavailable forms over time (Tveit et al. 2015; Brouns et al. 2022). In contrast, ED‐glycolysis was minimally expressed in fen but increased in bog microcosms over time (Figure 2A, right panel). Interestingly, both pathways were encoded in a significantly higher proportion of active MAGs in bog compared to fen (*p* = 0.00005) (Figure 2A, left panel). This inverse relationship between genetic potential and expression level suggests that substrate availability, rather than metabolic capacity, limits pathway activity. This resource‐dependent regulation aligns with ecological theory, as previous studies have demonstrated that microbial communities under resource limitation often maintain greater metabolic versatility while showing lower expression of specific pathways until appropriate substrates become available (Morrissey et al. 2017; Tian et al. 2020). The minimal expression of ED‐glycolysis in fen coupled with its increase in bog over time suggests that ED‐glycolysis may be a more energy‐efficient pathway under substrate‐limited conditions, allowing the bog microbial community to maximize energy yield from scarce resources.

For reductive pathways (Figure 2A, right panel), NADH‐recycling fermentations (ethanol and propionate) showed higher expression in fen microcosms and increased over time. Lactate fermentation decreased in fen but increased in bog microcosms between initial and final timepoints, suggesting different strategies for electron disposal under varying resource conditions. ATP‐producing pathways (acetate and butyrate fermentation) were similarly expressed in both systems. Despite lower expression levels, bog microcosms had significantly more MAGs encoding ethanol (*p* = 0.00005) and propionate (*p* = 0.02) fermentation, while fen had more MAGs encoding lactate fermentation (*p* = 0.0016). These patterns suggest a higher energetic state in fen microcosms, consistent with the greater abundance of labile compounds we observed in our metabolomic analysis. Here, energetic state refers to the cellular capacity for energy production and redox balance as supported by the availability of labile organic compounds. Previous studies have shown that NADH‐recycling pathways require sufficient energy resources to maintain, as they often involve energetically costly enzymes (Hoelzle et al. 2014; Kim and Gadd 2019). The increased expression of these pathways in fen microcosms, despite having fewer MAGs encoding them, indicates that substrate availability enables greater investment in redox balancing during active organic matter degradation.

We then examined the relationship between oxidative pressure (ED + EMP) and fermentation processes within individual MAGs. For clarity, community‐level analysis (Figure 2A) represents the aggregate expression of metabolic pathways summed across all microorganisms in each environment, while MAG‐level analysis examines patterns within each individual microbial genome, which represents a distinct population of closely related organisms. While Figure 2A displays community‐level expression patterns, we analyzed transcript abundance correlations between oxidative and reductive pathways at the individual MAG level, which showed distinct temporal patterns (Figure 2B). Statistical analysis of these correlations revealed that in the bog, the correlation coefficient increased from *r* = 0.39 to *r* = 0.63 over time, while in the fen, it decreased from *r* = 0.8 to *r* = 0.65.

**FIGURE 3:**
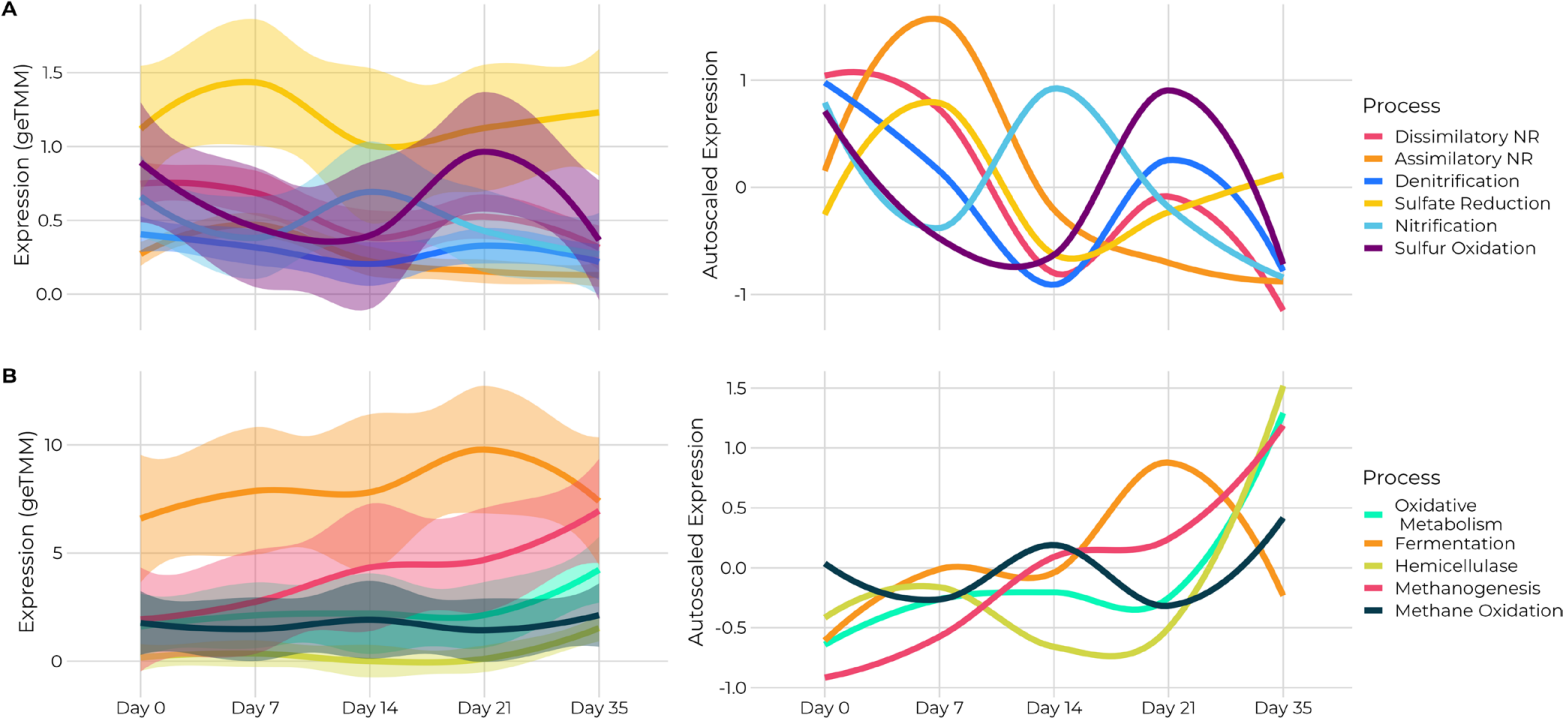
Community‐level transcription dynamics of different metabolic pathways over time in the fen microcosm. The left‐hand side of the graph shows actual transcription abundance (metaTMM normalized counts) representing the collective activity of the entire microbial community, while the right‐hand side shows all pathways centered and scaled for better comparison of temporal patterns. Colored bands around the actual transcription indicate standard error across all biological replicates. (A) Community‐level transcription of inorganic terminal electron transfer pathways, including reduction and oxidation processes. Reduction pathways include denitrification (dark blue), dissimilatory nitrate reduction (DNRA; pink), and assimilatory nitrate reduction (ANRA; orange), all of which fall under nitrate reduction, as well as sulfate reduction (yellow). Oxidation pathways include nitrification (light blue) and sulfur oxidation (purple). (B) Comparison of carbon metabolic processes, including fermentation (orange), oxidative metabolism (mint), hemicellulase activity (yellow‐green), methanogenesis (red), and methane oxidation (black). Oxidative metabolism represents the combined expression of Embden–Meyerhof–Parnas (EMP) and Entner–Doudoroff (ED) glycolytic pathways. These community‐level expression patterns reflect the aggregate response of the active microbiome to changing redox conditions during nutrient‐amended incubation.

While correlation coefficients alone cannot definitively demonstrate causality in metabolic coupling (Faust and Raes 2012; Weiss et al. 2016), contrasting temporal trends in these relationships, combined with our other gene expression data, suggest different metabolic regulation patterns. In the bog environment, the increasing alignment between glycolysis and fermentation gene expression across time points is consistent with previous observations that organisms under substrate limitation may coordinate these pathways more tightly as an efficiency strategy (Spaans et al. 2015; Flamholz et al. 2013). Conversely, the slight decrease in correlation observed in fen MAGs occurs alongside the increasing expression of alternative electron acceptor pathways shown later in figure 3, potentially representing diversification of electron flow (Conrad 2002; Bethke et al. 2011).

Examining expression patterns of individual MAGs complements our community‐level analysis by revealing genome‐level coordination that might be obscured in aggregate data. However, we acknowledge that correlation patterns can be influenced by various factors, including data distribution and outliers, and should be interpreted cautiously as part of our broader multi‐omics analysis rather than as standalone evidence of metabolic strategies.

Our results collectively demonstrate that substrate availability profoundly shapes microbial metabolism in permafrost‐affected peatland active layers, consistent with the metabolism‐centered redox framework. In this framework, oxidative processes necessitate balancing through increased reductive processes to maintain redox homeostasis (Flamholz et al. 2013; Spaans et al. 2015), a pattern that manifests differently in bog and fen environments as shown in our gene expression data (Figure 2). The lower degree of oxidative processes in the bog aligns with our earlier findings of reduced substrate availability compared to the fen. This observation underscores the direct link between DOM composition, substrate availability, and metabolic strategies, a relationship previously observed in similar peatland systems (Woodcroft et al. 2018; Singleton et al. 2018). These differences in substrate utilization alter the oxidative pressure within each system, which in turn influences the extent and nature of reductive metabolism.

### 3.3 Sequential Electron Acceptor Usage Maintains Redox Balance in Active Layer Peat From Fen Ecosystems

To explain the weakening relationship between oxidative and fermentative processes in active layer peat fen microcosms, we examined alternative electron acceptor pathways as potential electron sinks for maintaining redox balance, a strategy well documented in anaerobic environments (Bethke et al. 2011; Keller and Bridgham 2007). Community‐level metatranscriptomics analysis revealed coordinated patterns in terminal electron acceptor (TEA) usage over time (Figure 3A). Our transcriptomic data showed that from days 0–7, expression of genes involved in multiple nitrate reduction pathways increased. This included both denitrification genes (*narGHI*, *nirKS*, *nosZ*) and dissimilatory nitrate reduction to ammonium (DNRA) genes (*nirBD*, *nrfA*) (Table S5). The presence of expressed *nrfA* genes, which encode cytochrome c nitrite reductase that catalyzes the direct reduction of nitrite to ammonium in the DNRA pathway, indicates that at least some of the nitrate reduction occurred via DNRA rather than exclusively through denitrification. Between days 7 and 14, expression of both nitrate reduction pathways decreased as nitrification genes (*amoABC*, *hao*, and *narGH*) increased, suggesting that ammonia availability had increased, potentially from DNRA activity. From days 14–35, nitrification gene expression decreased while both denitrification and DNRA genes slightly increased. Similarly, sulfur oxidation increased from days 13–21 before declining, accompanied by increased sulfate reduction from days 14–35. These oscillating patterns suggest cyclic redox processes functioning as a mechanism for electron balance. While individual pathway changes at the community level were not statistically significant when evaluated in isolation, the consistent directional trends across multiple TEA pathways and the correlated patterns in nutrient uptake and utilization genes (Figure S2) provide strong support for the biological relevance of these coordinated redox balancing mechanisms rather than random fluctuations. Examining how these community‐wide TEA dynamics affected carbon metabolism (Figure 3B), we found clear evidence that alternative electron acceptors were compensating for the weakening fermentation pathway in the fen. Initial nitrate and sulfate reduction (days 0–7) coincided with moderate increases in oxidative metabolism and fermentation genes, providing electron sinks necessary for balancing redox status during carbon oxidation. When TEA reduction decreased (days 7–14), we observed a corresponding lag in oxidative metabolism and fermentation, further supporting the coupled relationship between TEA availability and carbon metabolism. As nitrification and sulfur oxidation declined while TEA reduction increased again (days 14–35), oxidative metabolism and hemicellulase expression rose sharply, demonstrating how alternative electron acceptors enabled enhanced substrate oxidation when fermentation alone was insufficient for redox balance.

These patterns directly support our hypothesis that alternative electron acceptors play a crucial role in maintaining redox balance in the fen. Increased oxidative processes are accompanied by proportional increases in nitrate and sulfate reduction, providing the necessary mechanisms to sink electrons generated during substrate oxidation. The temporary stall in oxidative metabolism during peaks in nitrification and methane oxidation suggests that the accumulation of reduced products affects the thermodynamic favorability of carbon degradation pathways.

Following the classical thermodynamic progression of electron acceptors, we examined methanogenesis dynamics in relation to other TEAs. While nitrate and sulfate reduction showed a second transcriptional peak in the latter half of the incubation, this peak was smaller than the initial one, potentially due to substrate limitations despite increased nutrient uptake systems. Both acetoclastic and hydrogenotrophic methanogenesis transcripts increased throughout the incubation (Figures 3B and S3), with methanogenesis becoming more prominent as other TEAs were depleted, which could have provided an additional electron sink when alternative TEAs were insufficient.

**FIGURE 4:**
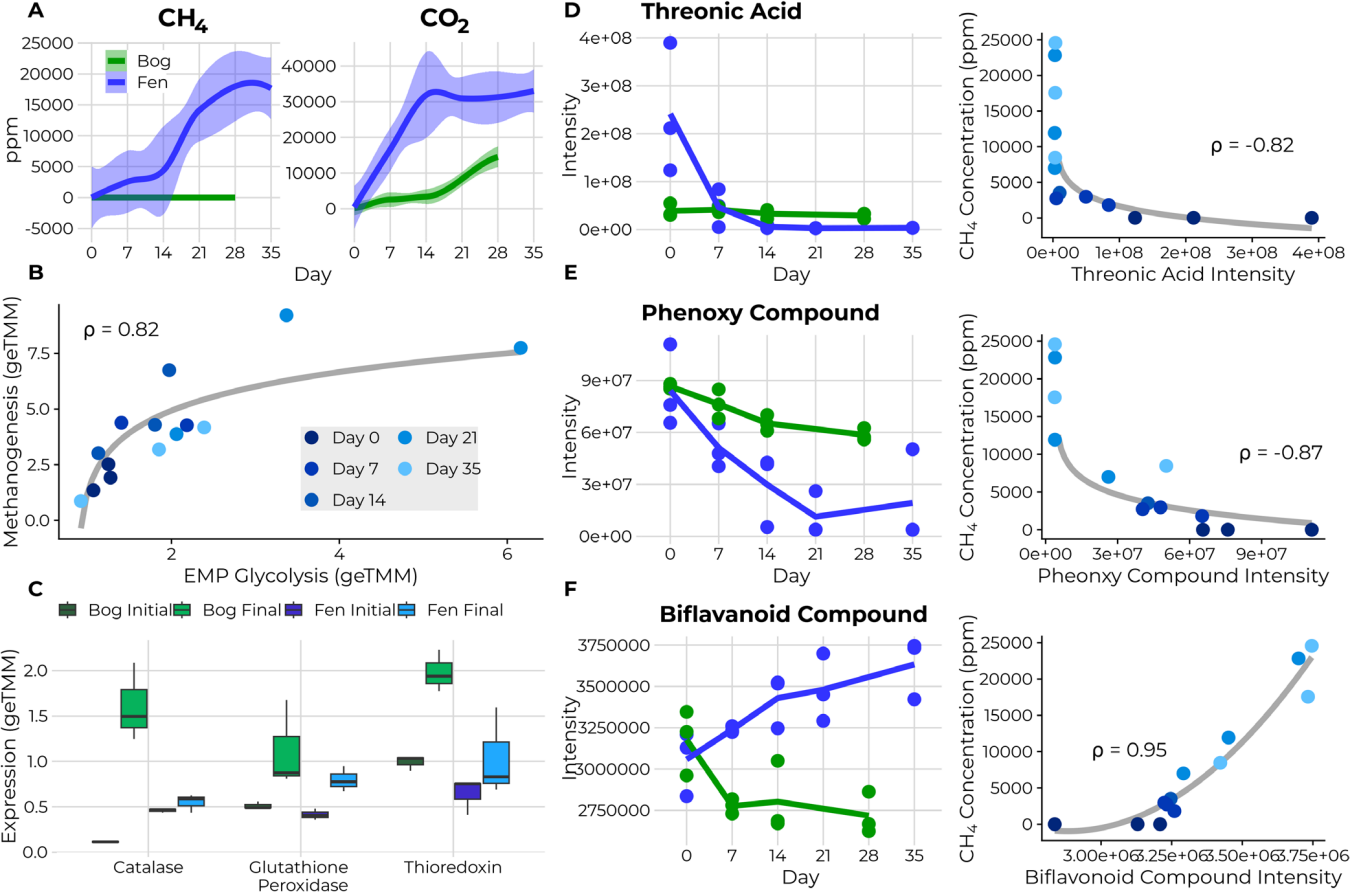
(A) Methane and carbon dioxide production measured from the bog (green) and fen (blue). (B) Relationship between transcription of EMP‐glycolysis and methanogenesis in the fen. (C) Expression of stress‐response genes in the bog compared to in the fen. (D–F) Tracking the fates of specific metabolites across time in the bog and fen (left‐hand) as well as their correlation with methane gas in the fen (right‐hand). Bog and fen initial refer to day 0, while bog final refers to day 28 and fen final refers to day 35.

Interestingly, we observed the greatest increase in fen CH_4_ concentration between days 14 and 21 (Figure 4A), coinciding with increased nitrate reduction and preceding increased sulfate reduction, suggesting competition between processes. This occurred despite no corresponding spike in methanogenesis gene expression but aligned with decreased methane oxidation during the same period. The slight decrease in oxidative metabolism during this time may have led to acetate accumulation, making carbon oxidation thermodynamically unfavorable until methanogenesis restored favorable conditions. This apparent decoupling between gene transcription and observed gas production illustrates the complex interplay of mechanisms involved in maintaining redox balance in peatland ecosystems.

As shown in Figure 4A, greenhouse gas concentration measurements revealed striking differences in production between bog and fen habitats at the ecosystem level. Fen microcosms produced substantially higher quantities of both CH_4_ and CO_2_ compared to bog microcosms throughout the incubation period. By day 35, CH_4_ concentrations in the fen reached approximately 20,000 ppm, while bog microcosms produced negligible methane, remaining below 500 ppm. Similarly, CO_2_ production was markedly higher in the fen, reaching around 30,000 ppm compared to approximately 10,000 ppm in the bog. These measurements provide direct quantification of the contrasting greenhouse gas profiles previously observed in field studies of permafrost‐affected peatlands (Holmes et al. 2022; Hodgkins et al. 2014).

The chemical composition differences we observed between bog and fen environments create distinct metabolic conditions that mechanistically explain these contrasting greenhouse gas emissions. Fen environments, with their higher abundance of readily oxidizable substrates, generate increased oxidative pressure that requires balanced reductive metabolism, including methanogenesis, as demonstrated by our gene expression data. This redox‐coupling mechanism provides a mechanistic understanding that bridges observed differences in organic matter chemistry with functional outcomes in carbon cycling (Fudyma et al. 2019; Conrad 2002). Our observations of higher oxidative metabolism in the fen and energy diversion toward stress responses in the bog further support the metabolism‐centered redox framework as an explanatory model for peatland carbon dynamics.

### 3.4 Metabolite Dynamics Drive Methanogenesis in Fen Ecosystems

To further investigate the relationship between substrate availability, oxidative metabolism, and methanogenesis, we employed Weighted Gene Co‐expression Network Analysis (WGCNA) to identify metabolites correlated with methane production. Metabolites negatively correlated with methane production were interpreted as contributing to methanogenesis, while those positively correlated were viewed as inhibiting methanogenesis. Due to limited annotations, ClassyFire was used to predict compound classes based on MS2 fragmentation patterns (Table S7). Choline, a tetraalkylammonium salt, was found to be negatively correlated with methane production, possibly by serving as a substrate for methylotrophic methanogenesis (Ellenbogen et al. 2023). Two compounds were predicted to be arylsulfonic acids, one as an alkanesulfonic acid, and one as a sulfobenzoic acid. Sulfonates, known to contribute to sulfate reduction (Kertesz 2000; Denger et al. 2012), may contribute to the initial sulfate reduction observed in the system. Multiple sugar‐like compounds were also found to be negatively associated with methane production, including allose, glucosaminitol, 2‐deoxy‐lyxo‐hexose, glucuronic acid, lyxonate, mannitol, threonic acid, and iduronate. In fact, multiple compounds were annotated as glucuronic acid and allose, suggesting multiple isomers of these sugars are present; however, we are unable to tell them apart using mass spectrometry. These easily degradable compounds may contribute to methanogenesis by increasing the oxidative pressure of the system, necessitating methanogenesis to maintain global redox balance and favorable conditions for upstream processes. To test this hypothesis, we examined the relationship between mapped gene expression for EMP glycolysis and mapped gene expression for acetoclastic and hydrogenotrophic methanogenesis in the fen, finding a strong positive correlation (rho = 0.82), further suggesting that increasing oxidative pressure must be balanced by reductive processes (Figure 4B). As the EMP‐glycolysis pathway generates more reducing equivalents (NADH) per glucose molecule compared to the ED pathway, it creates higher oxidative pressure that requires balanced electron‐accepting processes (Flamholz et al. 2013; Spaans et al. 2015; Conway 1992). When other terminal electron acceptors become limited, methanogenesis serves as a necessary electron sink to maintain redox homeostasis in environments with sufficient energy availability for EMP‐glycolysis, consistent with previous observations of metabolic coupling in anaerobic systems (Conrad 2002; Ungerfeld 2015).

Conversely, compounds positively associated with methane production (i.e., not consumed) were recalcitrant compounds, including flavan‐3‐ols, 3‐hydroxyflavonoids, 4′‐hydroxyflavonoids, and chromones, which have been shown to resist microbial degradation under anaerobic conditions (Wang et al. 2015; Romanowicz et al. 2015). Phloretin, daidzein, psoralen, orcinol‐dicarboxylic acid, and procyanidin B1, all phenolic compounds, were found to be positively correlated with methane production (rho ≥ 0.9).

Among metabolites negatively correlated with methane concentrations, 43 showed no significant difference in abundance between bog and fen microcosms, while 57 were significantly different: 35 more abundant in the fen and 22 in the bog. By contrast, for metabolites found to be positively correlated with methane concentrations (i.e., presumably not contributing to methanogenesis), 75 were non‐significantly different between the bog and fen, while 25 were significantly different, 22 of which were higher in the bog, while only 3 were higher in the fen. Furthermore, the compounds positively correlated with methane production had a mean NOSC of −0.054, while those negatively correlated with methane had a mean NOSC of 0.24, a significant difference (*p* = 0.001). This NOSC difference is mechanistically important because compounds with higher NOSC values are more oxidized and thus yield less energy when metabolized, making them less favorable substrates for microbial metabolism (LaRowe and Van Cappellen 2011). Conversely, compounds with lower NOSC values (more reduced) provide more electrons and energy during oxidation, making them preferentially consumed by microbes. The accumulation of high‐NOSC compounds during incubation therefore indicates selective utilization of more energy‐rich substrates, while the depletion of low‐NOSC compounds reflects their preferential consumption. While these compounds represent only a portion of the total organic matter pool, this difference in NOSC between compounds being consumed versus accumulated during incubation supports our hypothesis that differences in the utilization of specific organic matter compounds drive metabolic differences, which in turn affect methanogenesis.

We next further compared the fates of specific metabolites in the bog vs. the fen microcosms. Threonic acid, a sugar acid, was rapidly depleted in the fen (by day 14) while being both of lower abundance and degraded more slowly in the bog (Figure 4D). This compound (NOSC of 0.5) showed a −0.82 correlation with methane emissions in the fen. A phenoxy compound (NOSC of −2.67) declined more rapidly in the fen despite similar initial concentrations (Figure 4E). While phenoxy compounds can vary in their biodegradability depending on structure and environmental conditions (Fuchs et al. 2011; Boll et al. 2014), the more rapid depletion of this compound in the fen suggests differences in metabolic capabilities between the two environments. This compound was strongly negatively correlated with methane production (rho = −0.87). Consistent with this observation, we detected increased expression of aromatic compound degradation pathways, including phloroglucinol and benzoyl‐CoA pathways, in the fen compared to the bog (Figure S2). Lastly, we examined a bioflavonoid positively associated with methane production (NOSC of 0.96, rho = 0.95) (Figure 4F). This compound increased in abundance in the fen but declined in the bog, possibly due to differences in microbial degradation capacity in the fen and abiotic interactions in the acidic bog environment (Matsuo et al. 1984; Hättenschwiler and Vitousek 2000).

Our metabolite analysis demonstrates differential utilization patterns between bog and fen environments, with specific examples like threonic acid and aromatic compounds being more rapidly degraded in the fen. These observations, coupled with higher expression of carbon processing pathways in the fen, suggest more efficient carbon cycling in this environment compared to the bog. In contrast, the bog showed greater accumulation of certain compounds, particularly phenolics, which may contribute to slower carbon cycling (Freeman et al. 2001; Wang et al. 2015).

These contrasting metabolic environments align with the observed differences in greenhouse gas emissions between bog and fen ecosystems in our study. Although our experiments did not directly test the introduction of permafrost carbon, previous field studies have documented shifts from bog‐like to fen‐like conditions during permafrost thaw (Hodgkins et al. 2014; Hough et al. 2022). Our redox‐driven framework helps explain how such transitions might influence carbon cycling by demonstrating the metabolic mechanisms that link organic matter composition to greenhouse gas production. Future work will be needed to directly test how the introduction of previously frozen permafrost carbon affects these metabolic dynamics and subsequent greenhouse gas emissions as thawing continues in Arctic environments. More broadly, this redox‐driven framework provides a mechanistic basis for predicting how ecosystem transitions will alter greenhouse gas emissions as the Arctic continues to warm.

### 3.5 Energy in Active Layer Peat From Bog Ecosystems Is Diverted From Carbon Processing to Stress Response

Unlike the fen, the active layer peat bog appears to be thermodynamically limited by its DOM composition and struggles to degrade low‐NOSC compounds. Despite these limitations, both EMP and ED‐glycolysis occur within the bog but do not substantially contribute to methanogenesis as seen in the fen. This suggests energy in peatland bog ecosystems is redirected toward alternative functions.

Metabolomics analysis revealed higher phenolic compound abundance in the active layer peat bog compared to the fen (Figure 1B). These polyphenols not only limit substrate availability but may also generate stress conditions. In oxic systems, polyphenols can generate ROS at potentially bactericidal levels (Fathima and Rao 2016). Although our nutrient‐amended bog incubations were anoxic, several lines of evidence suggest oxidative stress: (1) catalase expression indicates hydrogen peroxide presence; (2) previous studies have shown phenol peroxidase activity in anoxic deep peat; and (3) hydroxy‐radical formation accelerates in acidic environments (Zhao et al. 2021). Brief exposure to oxygen during the nutrient‐amended incubation setup may have initiated these reactions, similar to fluctuating water tables in natural bogs. The acidic pH characteristic of bogs, often resulting from organic acid accumulation such as acetate (Hines et al. 2008), further stresses the microbial community. Research has shown that microbes respond to acid stress by inducing oxidative stress genes (Bruno‐Bárcena et al. 2010; Schellhorn and Stones 1992). Notably, the ED‐glycolysis pathway, which was both more highly encoded and expressed in bog microbes, has been shown to confer increased oxidative stress resistance through direct NADPH supply (Klingner et al. 2015). The higher prevalence of ED‐pathway genes suggests long‐term selective pressure toward stress resistance in the bog environment.

To test whether bog microbes divert energy toward stress response, we compared expression of oxidative stress genes between active layer peat bog and fen microcosms (Figure 4C) (Bengtsson‐Palme et al. 2014). Catalase, which transforms hydrogen peroxide to oxygen and water, showed higher expression in the bog. Similarly, glutathione peroxidase and thioredoxin, which use NADPH to reduce disulfide bonds caused by oxidative stress, were more highly transcribed in the bog compared to the fen. These results support our hypothesis that bog microbes redirect energy toward cellular maintenance at the expense of carbon processing and methanogenesis. Previous studies have shown that under acid stress, bacteria increase glycolytic metabolism but decrease biomass synthesis efficiency (Even et al. 2003).

Within our redox framework, these findings suggest that energy in active layer peat from permafrost‐affected bog ecosystems is prioritized for cellular maintenance and stress response rather than biomass synthesis and recalcitrant compound degradation. This redirection slows organic matter degradation, reducing oxidative pressure and preventing methanogenesis. Additionally, the high polyphenol content in bogs may serve as an electron acceptor (Gunina and Kuzyakov 2022), allowing microbes to balance redox status through anaerobic respiration on humic substances rather than producing hydrogen needed for methanogenesis (Obradović et al. 2024; Wilson et al. 2017). Interestingly, although anaerobic humic‐acid respiration expression is higher in the fen than in the bog (Figure S4), the fen's greater oxidative pressure appears to exceed the electron‐accepting capacity of this pathway.

## 4 Discussion

Using a metabolism‐centered redox framework and multi‐omics analyses of active layer peat microcosms, we provide the first empirical test demonstrating how organic matter composition mechanistically drives greenhouse gas production in thawing permafrost‐affected ecosystems. Our results support the hypothesis that examining redox processes through metabolic pathways, rather than through direct geochemical measurements, provides unique insights into microbial regulation of carbon cycling, particularly revealing how energy allocation strategies dynamically respond to substrate availability and quality. Our findings reveal a fundamental tradeoff in carbon processing: active layer peat from fen environments, enriched in easily degradable substrates, creates high oxidative pressure that drives methanogenesis, while bog environments force energy allocation toward stress responses, thereby limiting methane production. This mechanistic understanding extends beyond previous correlative studies of organic matter changes during permafrost thaw (AminiTabrizi et al. 2020; Hodgkins et al. 2014; Hough et al. 2022; Wilson et al. 2016, 2022) and demonstrates the potential for our framework to provide a predictive framework for how ecosystem transitions will alter carbon‐climate feedbacks.

### 4.1 Energy Allocation and Ecosystem Function

The most significant insight from our study is the evidence for an energy‐allocation hypothesis in carbon‐limited ecosystems. This energy‐allocation hypothesis represents a novel concept in understanding microbial ecology in permafrost‐affected peatlands and expands upon previous work. The higher transcription of stress‐response genes and ED‐glycolysis in the bog environment indicates energy redirection towards cellular maintenance rather than methanogenesis. This metabolic strategy has broader implications, as it may explain observations from other studies where the addition of humic acids to peat soil decreased methane production (Keller et al. 2009), potentially through the redirection of energy towards cellular maintenance and electron sinking into humic degradation and anaerobic respiration. Our findings also align with studies showing that the addition of labile carbon to deep peat results in increased GHG production and reinvestment of energy into aromatic compound degradation (Rajakaruna et al. 2024). Others have shown that acidic pHs and polyphenols can limit methanogen activity, and these processes may indeed be at play in our microcosms (McGivern, Ellenbogen, et al. 2024; Ye et al. 2012).

An important consideration is whether the lower methanogenesis in bog environments results from reduced methanogen abundance (0.3% of transcripts in bog vs. 2% in fen) or from metabolic constraints on existing methanogens. While our data show that methanogen transcript abundance correlates with methane production patterns, this relationship could reflect either (1) selective pressure against methanogens in stressful bog conditions, leading to lower community representation, or (2) metabolic suppression of methanogen activity due to energy allocation toward stress responses, as our framework suggests. These explanations are not mutually exclusive—both community filtering and metabolic constraints likely contribute to reduced methanogenesis in bog environments. Our energy‐allocation hypothesis provides a mechanistic explanation for how environmental stressors could simultaneously reduce methanogen fitness (leading to lower abundance) and suppress methanogenic activity in remaining populations through energy redirection toward cellular maintenance.

The contrasting community composition patterns we observed align with previous studies at Stordalen Mire. *Acidobacteriota* dominated metabolic activity in bog environments, while fen systems showed more balanced expression across multiple phyla. Woodcroft et al. (2018) reported similar community patterns, and our findings extend this understanding by demonstrating how these community differences manifest in specific metabolic adaptations rather than simply reflecting taxonomic preferences. This pattern demonstrates how environmental constraints shape both community composition and metabolic strategies in these contrasting ecosystems.

### 4.2 Community Adaptation to Environmental Constraints

The contrasting community composition and gene expression patterns we observed align with previous studies at Stordalen Mire. *Acidobacteriota* dominated both transcript recruitment and expression in active layer peat bog microcosms (64% of transcripts), while the fen showed more balanced expression across multiple phyla. Woodcroft et al. (2018) reported similar patterns, with *Acidobacteriota* dominating metabolic activity in bog environments. Our findings extend this understanding by demonstrating how these community differences manifest in specific metabolic adaptations rather than simply reflecting taxonomic preferences. The prevalence of ED‐glycolysis in bog communities represents a long‐term adaptive strategy for stress resistance rather than merely a response to substrate limitation.

### 4.3 Redox Cycling and Ecosystem Resilience

The terminal electron acceptor dynamics we observed in active layer peat fen microcosms demonstrate the complex interplay between carbon oxidation and reduction pathways. The oscillating patterns between nitrate reduction, nitrification, sulfate reduction, and sulfur oxidation reflect how microbes dynamically respond to changing substrate and electron acceptor availability. This cyclical behavior aligns with Conrad's (2002) observations that seemingly competitive metabolic pathways can coexist in natural systems. These patterns suggest that microbial communities maintain resilience through flexible resource allocation, adapting to changing redox conditions while maintaining system‐level carbon processing. The eventual progression to methanogenesis after depletion of more energetically favorable electron acceptors confirms traditional thermodynamic understanding (Heimann et al. 2010) while extending it through our redox framework, which appears to successfully integrate metabolic pathway analysis with redox dynamics. The apparent decoupling between methanogenesis gene expression and observed methane production highlights the complexity of biogeochemical cycling and suggests additional factors beyond transcriptional regulation. Our metabolomic analyses revealed specific compounds that may contribute to or inhibit methanogenesis. Choline, which can serve as a substrate for methylotrophic methanogenesis (Ellenbogen et al. 2023), negatively correlated with methane production, along with multiple sugar‐like compounds, including threonic acid, glucosaminitol, and allose. These easily degradable substrates likely increase oxidative pressure, driving methanogenesis to maintain redox balance. The significant difference in NOSC values between compounds positively and negatively correlated with methane production (*p* = 0.001) provides direct evidence linking organic matter composition to methanogenic activity. Specifically, compounds with lower NOSC values that were consumed during incubation represent more reduced, energy‐rich substrates that generate excess electrons during oxidative metabolism (LaRowe and Van Cappellen 2011). In contrast, compounds with higher NOSC values (mean = 0.24) that accumulated represent more oxidized, energy‐poor substrates that are less favorable for microbial metabolism. This selective consumption pattern demonstrates that substrate oxidation state directly influences microbial substrate preference and, consequently, the magnitude of electron flow that must be balanced through methanogenesis.

### 4.4 Implications for Carbon‐Climate Feedbacks

As permafrost continues to thaw over the coming decades, understanding the mechanistic links between organic matter quality and greenhouse gas production becomes increasingly critical. Our findings suggest that transitions from bog‐like to fen‐like conditions during thaw could accelerate methane emissions through two mechanisms we identified: (1) increasing substrate availability that drives oxidative pressure, as evidenced by our observation that fen systems with higher sugar content and lower NOSC values showed increased methane production and glycolysis gene expression, and (2) alleviating stress conditions that otherwise divert energy from methanogenesis, as demonstrated by bog samples showing higher stress‐related gene expression and lower methanogenic activity despite thermodynamically favorable conditions. These experimental findings provide mechanistic insight into how the carbon composition changes during thaw (liberation of ancient carbon and input of new labile carbon from enhanced plant productivity) translate into altered greenhouse gas production through shifts in microbial energy allocation strategies. These ecological transitions may occur over varying timescales, from years to decades depending on local hydrology and warming rates, potentially creating hotspots of accelerated methane emissions/production that current climate models may not adequately capture.

This mechanistic understanding provides predictive power beyond correlative approaches, offering potential intervention points for managing methane emissions across ecosystems. For example, introducing compounds that increase cellular stress might suppress methanogenesis in vulnerable settings, though such interventions would require careful ecological consideration.

### 4.5 Limitations and Future Directions

An important limitation of our study is the ability to definitively establish causation versus correlation in the relationships we observed. While our multi‐omics approach provides compelling evidence for the mechanistic links between organic matter composition, microbial metabolism, and greenhouse gas production, several key relationships warrant careful interpretation.

The strong correlation between oxidative pathways (glycolysis) and reductive pathways (methanogenesis) in the fen (rho = 0.82) suggests a functional connection but does not conclusively prove that increased oxidative metabolism directly causes increased methanogenesis. Multiple intermediary processes could influence this relationship, including interspecies metabolite transfer, thermodynamic constraints, or community‐level adaptations. Similarly, while we observed increased stress‐response genes coinciding with lower methanogenesis in the bog, we cannot definitively conclude that stress responses directly cause reduced methane production. Both could be independent responses to the acidic, phenolic‐rich environment characteristic of bog ecosystems.

Our study focused on the active layer peat (9–19 cm depth) overlying permafrost rather than the permafrost itself. While our findings provide important insights into carbon cycling in permafrost‐affected peatlands, the behavior of carbon released directly from thawing permafrost may involve additional processes not captured in our experiments. As permafrost continues to thaw, understanding these mechanistic links between organic matter quality and greenhouse gas production becomes increasingly critical. Our metabolism‐centered redox framework and energy‐allocation hypothesis provide new approaches for predicting how ecosystem transitions will alter carbon‐climate feedbacks. Moreover, these insights suggest potential intervention points for managing methane emissions across systems, from thawing permafrost to agricultural settings.

Our metabolism‐centered redox framework, as tested in this study, provides new insights into previous observations. For instance, studies have shown that sulfate addition can have complex, concentration‐dependent effects on methanogenesis. AminiTabrizi et al. (2023) observed that while sulfate inhibits methanogenesis at low concentrations through competitive inhibition between methanogens and sulfate‐reducing bacteria, the relationship becomes more complex at higher concentrations (5 mM). Traditional thermodynamic perspectives that focus solely on competition between methanogens and sulfate reducers cannot fully explain these observations. Our redox framework suggests an alternative explanation: increased sulfate availability at higher concentrations could stimulate greater oxidative metabolism, which, after sulfate reduction is exhausted, must be balanced by increased methanogenesis—similar to the sequential electron acceptor usage patterns we observed in our study.

Metabolite correlations with methane production also require careful interpretation. The observed negative correlations between certain compounds (e.g., threonic acid, phenoxy compounds) and methane production do not necessarily indicate direct mechanistic involvement in methanogenesis. These relationships could reflect (1) compounds being consumed as carbon sources, indirectly fueling methanogenesis through intermediate metabolites; (2) compounds serving as indicators of broader metabolic processes without direct involvement; or (3) coincidental depletion patterns driven by separate microbial activities. The statistical correlations presented in our study should therefore be viewed as supporting evidence for potential mechanisms rather than definitive proof of causal relationships. This interpretive caution is further reinforced by methodological considerations in our metatranscriptomics analysis, where samples were processed at different facilities using different rRNA depletion kits, potentially introducing technical variation despite rigorous computational normalization approaches including geTMM normalization, consistent read processing with bbduk, standardized mapping parameters, and strict filtering criteria. To strengthen mechanistic understanding beyond correlations, future work could employ isotopic labeling to trace carbon flow through specific pathways, use selective inhibitors to perturb key processes, or develop targeted genetic manipulations in model organisms representing key functional groups. Time‐resolved experiments with higher temporal resolution might also better capture the sequence of metabolic events, helping distinguish cause from effect in the complex network of microbial interactions.

While our findings provide new insights into the relationship between organic matter composition and microbial metabolism, several questions remain unanswered and warrant further investigation. The acidic pH of the bog is believed to be driven in part by a buildup of organic acids from fermentation, and the values from the literature suggest acetate concentrations in the bog are in millimolar quantities, while the fen exists in micromolar quantities (Hines et al. 2008). From a redox perspective, this acetate accumulation in the bog might indicate a bottleneck in electron transfer to methanogens, possibly related to the energy allocation towards stress responses we observed. Despite this discrepancy in acetate levels, acetoclastic methanogenesis is typically greater in the fen compared to the bog (Figure S3), presenting a stark contrast between the two systems (Ellenbogen et al. 2023). The exact mechanism linking acetate formation to acetate degradation by methanogens has still yet to be elucidated and is paramount to understanding how increased oxidative pressure translates to methanogenesis. Indeed, mechanisms such as direct interspecies electron transport (DIET) might serve as important conduits for electron transfer; however, we detected no transcripts of Geobacter, a well‐known DIET microbe, in our microcosms. Another outstanding question is the source of oxidative stress for bog microbes. Bogs are known to be anoxic environments, and our microcosms were kept anaerobic. Polyphenols can non‐enzymatically form ROS, but this is only known to occur under oxic conditions (Fathima and Rao 2016). Some evidence shows that acidic stress, as observed in bog conditions, can elicit similar molecular responses from microbes (Bruno‐Bárcena et al. 2010; Schellhorn and Stones 1992). Potentially, the molecular oxygen formed by catalase could react with the phenols present to form ROS, but this mechanism remains unproven.

In summary, our first empirical test of a metabolism‐centered redox framework provides valuable insights by integrating multiple lines of evidence across different analytical platforms, despite the inherent limitations of field‐based ecological studies. The consistent patterns observed across metagenomics, metatranscriptomics, and metabolomics suggest robust underlying mechanisms governing carbon processing in these contrasting environments. Our results demonstrate that this approach moves beyond simple correlations by situating observed relationships within a theoretical framework that explains how organic matter quality could mechanistically influence microbial metabolism through energy allocation strategies. This balanced assessment of causation versus correlation strengthens rather than diminishes the significance of our findings, as it acknowledges the complexity of natural systems while still advancing our conceptual understanding of how organic matter composition influences greenhouse gas emissions in permafrost‐affected peatland ecosystems.

This study provides compelling evidence for associations between organic matter composition and microbial metabolism, generating testable hypotheses for future experiments examining redox potential within ecosystems. Our findings advance understanding of current carbon cycling processes and provide a mechanistic foundation for predicting ecosystem responses to continued Arctic warming, with potential applications for methane management strategies across diverse anaerobic systems.

## Author Contributions

**John A. Bouranis:** conceptualization, data curation, formal analysis, visualization, writing – original draft. **Bridget B. McGivern:** data curation, investigation, writing – review and editing. **Ghiwa Makke:** software, visualization, writing – review and editing. **Sophie K. Jurgensen:** data curation, writing – review and editing. **Samantha H. Bosman:** data curation, writing – review and editing. **Brooke Stemple:** data curation, writing – review and editing. **Jeffrey P. Chanton:** resources, writing – review and editing. **Kelly C. Wrighton:** conceptualization, funding acquisition, resources, writing – review and editing. **Malak M. Tfaily:** conceptualization, funding acquisition, investigation, resources, writing – original draft, writing – review and editing.

## Conflicts of Interest

The authors declare no conflicts of interest.

## Supporting information


**Figure S1:** gcb70390‐sup‐0001‐Figures.pdf.

**Table S1:** gcb70390‐sup‐0002‐TableS1.xlsx.

**Table S2:** gcb70390‐sup‐0003‐TableS2.xlsx.

**Table S3:** gcb70390‐sup‐0004‐TableS3.xlsx.

**Table S4:** gcb70390‐sup‐0005‐TableS4.xlsx.

**Table S5:** gcb70390‐sup‐0006‐TableS5.xlsx.

**Table S6:** gcb70390‐sup‐0007‐TableS6.xlsx.

**Table S7:** gcb70390‐sup‐0008‐TableS7.xlsx.

## Data Availability

The data and code that support the findings of this study are available on OSF at https://doi.org/10.17605/OSF.IO/YEM24 and GitHub at https://github.com/tfaily‐lab/Bog_Fen_Redox. Metatranscriptomics data are available at NCBI under BioProjectID PRJNA386568.
