# Supplementary material for: Metabolic Redox Coupling Controls Methane Production in Permafrost‐Affected Peatlands Through Organic Matter Quality‐Dependent Energy Allocation
Source: Glob Chang Biol. 2025 Aug 7;31(8):e70390. doi: 10.1111/gcb.70390 (PMC12329716; doi:10.1111/gcb.70390)
Supplement: Supplementary file 1 — Figure S1: gcb70390‐sup‐0001‐Figures.pdf. [file GCB-31-e70390-s001.pdf]

Title: Metabolic Redox Coupling Controls Methane Production in Permafrost-Affected Peatlands through Organic Matter Quality-Dependent Energy Allocation

Running title: Organic Matter Shapes Peatland Methane

List of Authors: John A Bouranis[1], Bridget B McGivern[2,3], Ghiwa Makke[1], Sophie K Jurgensen[2], Samantha H Bosman[4], Brooke Stemple[2], Jeffrey P Chanton[4], Kelly C Wrighton[2], Malak M Tfaily[1,5*]

List of Author's ORCID #s :
John A. Bouranis – 0000-0002-5533-7570
Bridget B. McGivern – 0000-0001-9023-0018
Kelly C. Wrighton – 0000-0003-0434-4217
Malak M. Tfaily – 0000-0002-3036-2833

Institutional affiliations:
1) University of Arizona, Department of Environmental Science
2) Colorado State University, Department of Soil and Crop Sciences
3) UW Eau Claire, Department of Chemistry and Biochemistry
4) Florida State University, Department of Earth Ocean and Atmospheric Science.
5) University of Arizona, Bio5 Institute

***Corresponding author:**
Department of Environmental Science, The University of Arizona, Tucson, AZ, USA
Bio5 Institute, The University of Arizona, Tucson, AZ, USA
tfaily@arizona.edu

Five supplementary figures are included that provide additional detail on the molecular mechanisms that were discovered in our permafrost research.

Figure S1 shows the phylogenetic diversity and activity of microbial communities - essentially which microorganisms were present and actively working in bog and fen environments across the study period.
Figure S2 demonstrates the expression of key nutrient transport genes - specifically nitrogen and sulfur transport pathways that are critical for ecosystem function and stability.
Figure S3 focuses on methanogenesis pathways - the biological processes by which methane is produced, which is crucial for understanding how these ecosystems contribute to greenhouse gas emissions.
Figure S4 examines polyphenol degradation genes - these are the pathways by which complex plant compounds are broken down, including our key metabolite catechin that served as the early warning signal.

Figure S5 shows alternative electron sinks - essentially the backup energy pathways that are used by microorganisms when their primary systems are disrupted.

Seven supplementary tables are attached; however, Table S4 is available on OSF https://doi.org/10.17605/OSF.IO/YEM24 due to its size.

Summary of tables attached as Excel files:

S1: Total gas production data - the sum of gaseous and dissolved phases represents the total production of each gas.
S2: Metabolomics data is provided in Supplementary Table S2.
S3: Raw bog and fen metatranscriptome and metagenomic reads.
S4: Metatranscriptome mapping data (available on OSF due to file size).
S5: Pathway analysis results are presented in Supplementary Table S5.
S6: Chemical class enrichment analysis.
S7: ClassyFire compound classification based on MS2 fragmentation patterns.

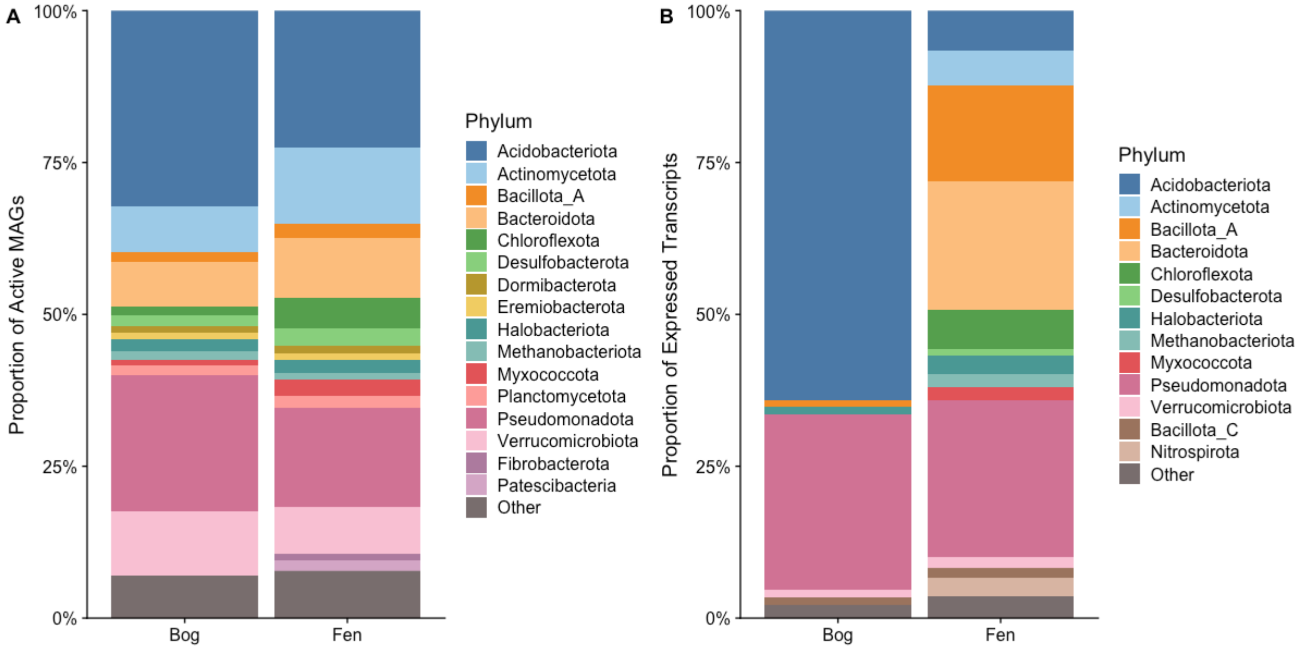

Figure S1: A) Bar plot showing the phylogenetic diversity of active MAGs which recruited transcripts in and bog and fen across all timepoints. Phyla which constituted less than 1% of total active MAGs were designated as “Other”. B) Bar plot showing the relative contribution of each phylum to total expressed transcripts. Phyla which constituted to less than 1% of transcript expression were designated as “Other”.

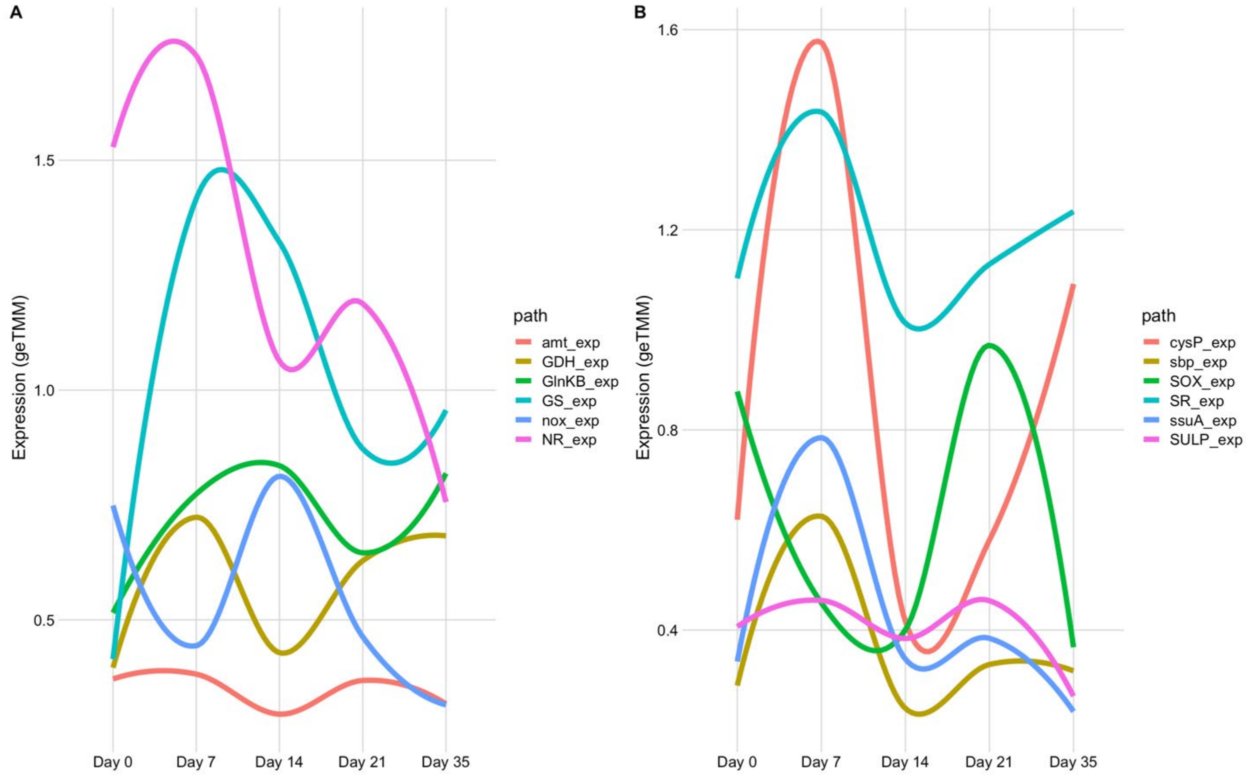

Figure S2: Expression of A) nitrogen transport genes and B) sulfur transport genes. amt: ammonium transporter; GDH: glutamate dehydrogenase; GS: glutamine synthase; glnKB: nitrogen regulatory protein p-II; NR: Nitrate reduction; nox: Nitrification; cysP: sulfate/thiosulfate transport system substrate-binding protein; sbp: sulfate/thiosulfate transport system substrate-binding protein; SULP: sulfate permease; ssuA: sulfonate transport system substrate-binding protein; SR: sulfate reduction; SOX: sulfur oxidation.

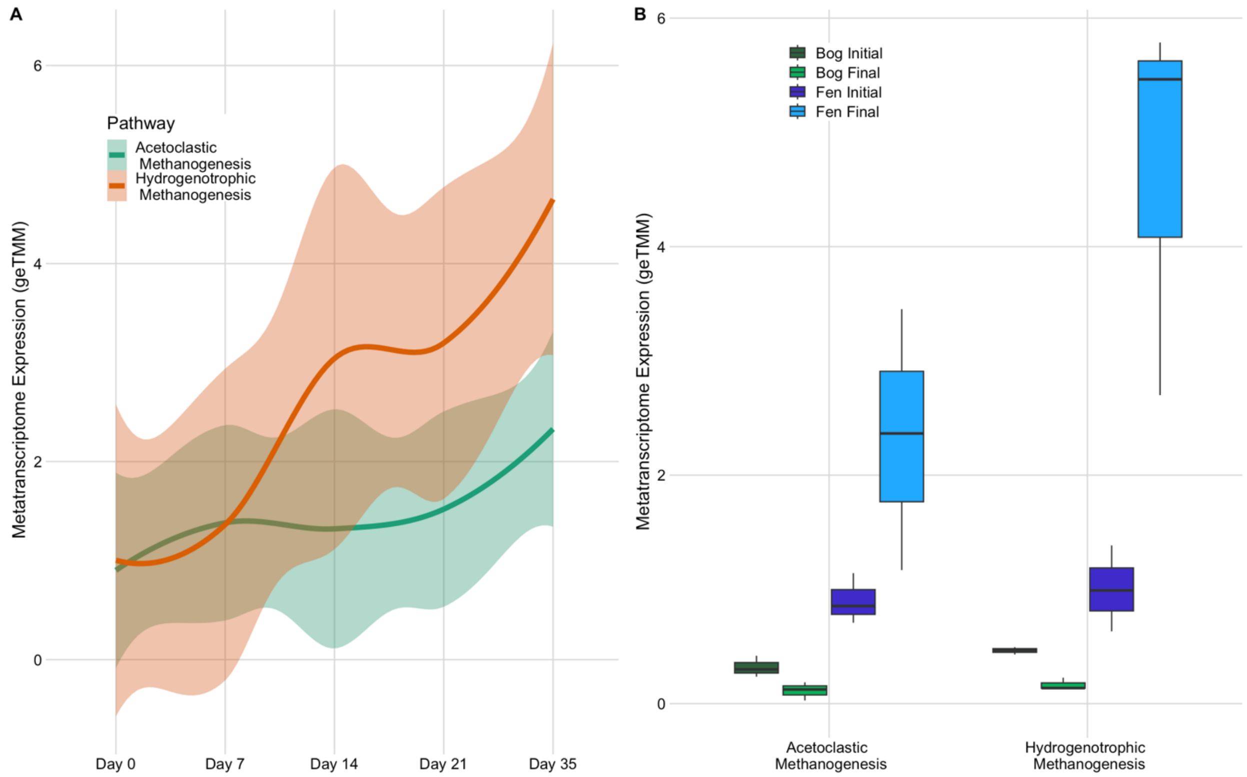

Figure S3: A) Expression of acetoclastic and hydrogenotrophic methanogenesis pathways in the fen microcosm over time. B) Expression of acetoclastic and hydrogenotrophic methanogenesis pathways in the bog and fen microcosms at the beginning and end of the nutrient-amended incubations.

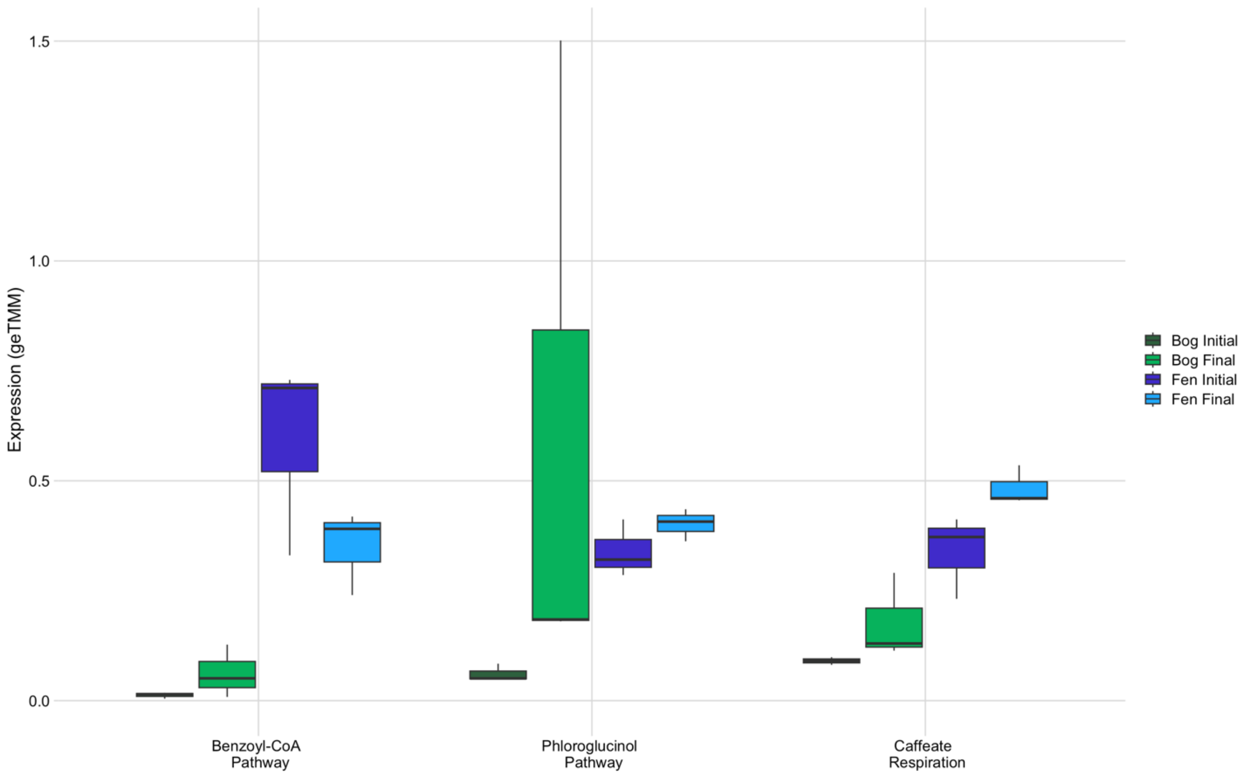

Figure S4: Expression of polyphenol degradation and respiration genes in the bog and the fen. Bog and fen initial refer to day 0 while bog final refers to day 28 and fen final refers to day 35.

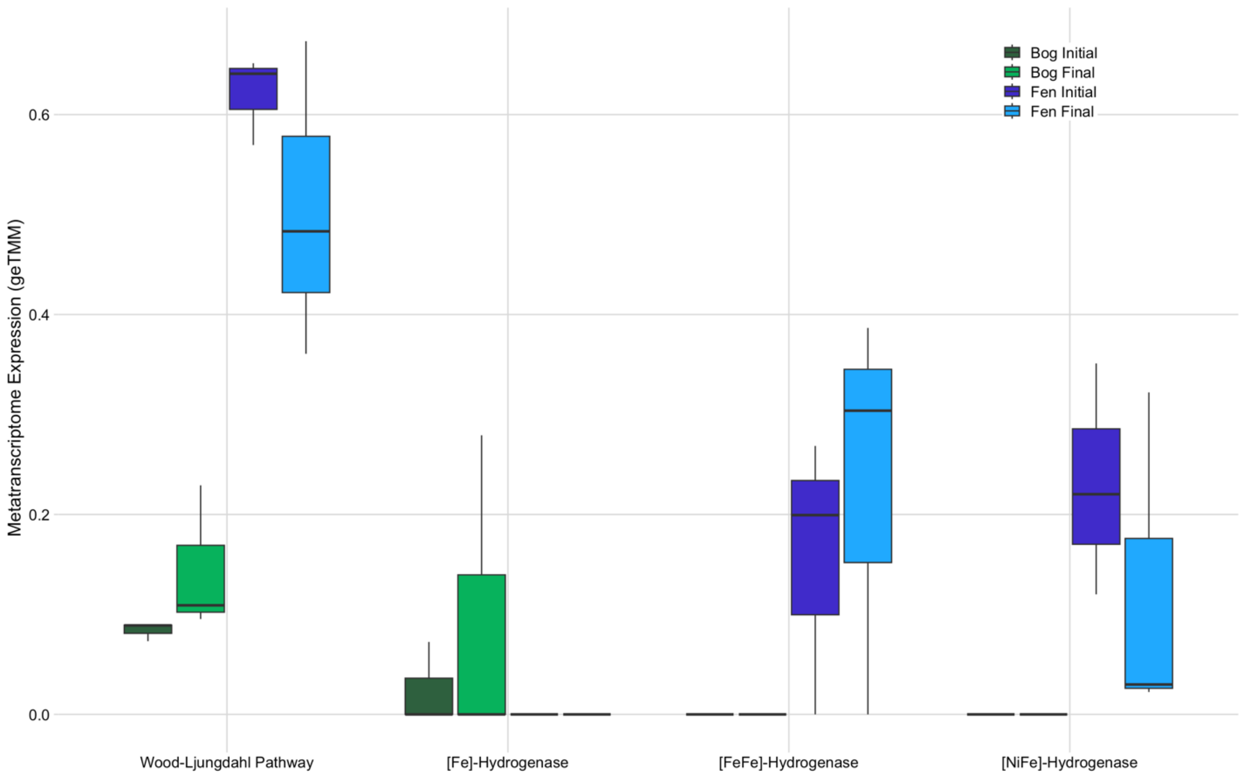

Figure S5: Expression of alternative electron sinks in the bog and fen microcosms and the beginning and end of nutrient-amended incubations.
